# Synthesis and performance evaluation of adsorbents derived from sewage sludge blended with waste coal for nitrate and methyl red removal

**DOI:** 10.1038/s41598-022-05662-5

**Published:** 2022-01-31

**Authors:** John Longo Masengo, Jean Mulopo

**Affiliations:** School of Chemical and Metallurgical Engineering, University of Witwatersrand, P/Bag 3, Wits, Johannesburg, 2050 South Africa

**Keywords:** Environmental sciences, Chemistry, Engineering

## Abstract

Low-cost adsorbents were synthesized using two types of sewage sludge: D, which was obtained during the dissolved air flotation stage, and S, which was a mixture of primary and secondary sludge from the digestion and dewatering stages. The sewage sludge was mixed with waste coal before being activated with potassium hydroxide (KOH) and oxidized with ammonium persulfate (APS). The nitrate and methyl red removal capacities of the synthesized adsorbents were evaluated and compared to those of industrial activated charcoal. The oxidation surface area of adsorbents derived from sludge S shrank by six fold after modification i.e., from 281.72 (unoxidized) to 46.573 m^2^/g for the oxidized adsorbent with a solution of 2M ammonium peroxydisulfate, while those derived from D only varied narrowly from 312.72 to 282.22 m^2^/g, but surface modification had no effect on inorganic composition in either case. The adsorption of nitrate and methyl red (MR) was performed in batch mode, and the removal processes followed the pseudo second order kinetic model and the Langmuir isotherm fairly well. The adsorption capacities of nitrate and MR were higher at pH = 2 and pH = 4, respectively.

## Introduction

Sewage sludge is an unavoidable by-product of wastewater treatment plants, with treatment costs accounting for 25–65% of the overall operating costs^1^. South Africa's population has gradually increased over the last two decades, rising from 48.8 million in 2005 to 51.58 million in 2010 to 57.7 million in 2018, with more than half of the country’s population now residing in densely populated urban areas^2^. Rapid population growth, combined with rapid urbanization, has put additional pressure on existing wastewater treatment plant (WWTP) facilities, resulting in increased sewage sludge production. Several lines of evidence demonstrate that dumping waste sludge in landfills is not a long-term sewage sludge management solution. Despite the risk of soil and subsoil contamination, nearly 80% of wastewater treatment plants continue to discharge sewage sludge at designated sites^3^.

The South African Department of Water Affairs and Forestry has identified five potential applications for sewage sludge: agriculture (fertilizer), on-site and off-site land application (within and outside WWTP boundaries), thermal management activities (e.g. full or partial combustion of organic solids by incineration), and benign land application^3^. The use of sewage sludge fertilizers is restricted due to the difficulties in meeting quality requirements (faecal coliform cap of 10000 CFU/g dry, Helminth Ova maximum of one viable ova/g dry, pollution)^3^. As a result of these restrictions, large quantities of untreated sludge have accumulated on open fields. South Africa produces 673360 metric tons of sewage sludge annually, of which only about 19% is recycled and the rest is landfilled, necessitating the development of innovative approaches to reduce sewage sludge emissions^2^. Landfilling as a wastewater treatment residue management alternative poses significant environmental concerns about bacteria, heavy metals, and trace organic pollutants; the same conclusion applies to traditional sea dumping and forestry, both of which are deemed unsustainable^4^.

Due to the high cost of industrial activated carbons, research on activated carbon materials extracted from bio-waste, such as olive stones, lemon grass, wall nutshells and pine cones has taken center stage^5–8^ and among these, activated carbon extracted from sewage sludge (SBAC) has shown promise in the adsorption of inorganic elements such as Ni, Cu, Pb, Cd and Hg^1,9–13^.

However, due to the inorganic composition of raw sewage sludge, SBAC has a low surface area, and increasing porosity by adding richer carbonaceous such as coal, bagasse, and coconut shell may be preferable^7,9,11,14,15^. Additionally, in South Africa, coal combustion accounts for 92% of electricity generation. Waste coal is disposed of as tailings after the coal beneficiation process and the separation of less coarse coal from washing and fines particles by screening. South Africa’s waste coal stockpile is estimated to be over 1.5 billion tons and is growing at a rate of 60 million tons per year. The stockpiling of waste coal has a detrimental influence on the environment, as it results in spontaneous combustion, the loss of usable land area, and the emission of hazardous substances. As a result, alternative solutions for waste coal beneficiation must be explored. On the other hand, The use of HCl and NaOH, HNO_3_ and N,N-Dimethylformamide for the oxidation of SBAC in liquid phase has been reported to increase their adsorption potential by adding surface functionalities groups as shown in Table 1^9,16–18^. An in-depth analysis of the existing body of works, on the other hand, exposes knowledge gaps, such as the fact that very few studies take into account the specific chemical composition of the source materials used. The synthesis of activated carbon from sewage sludge has traditionally been oriented toward particular applications and thus has been more focused on results than on understanding the intrinsic structure within the materials (Table 1). In particular, understanding the adsorption of certain inorganic compounds in aqueous solution by activated carbon requires knowledge of the chemical structure of the activated carbon surface, i.e. determining how the chemical structure of the activated carbon surface is oxidized during the chemical activation process in order to achieve the optimal porous surface. When the source material has been chemically treated, the surface of the activated carbons is normally filled with oxygenated sites and probably amine sites which result in three types of oxides on the surface: acidic, basic, and neutral. Acidic sites increase the hydrophilicity of activated carbon, lower the pH in aqueous suspension, and increase the negative charge density at the surface, while simple sites are primarily of the Lewis form and are associated with -rich regions located at the basal planes^19^. Additional benefits can be obtained by oxidizing activated carbon after the activation phase to induce the formation of oxygen complexes. The oxidation process raises the oxygen content by lowering the electronic density of the basal planes, which lowers the basicity at the surface^19–21^. The oxidation of activated carbon extracted from sewage sludge blended with coal using ammonium peroxydisulfate [(NH_4_)_2_S_2_O_8_] is investigated in this paper. In general, the oxidation process is intended to result in the formation of carboxylic sites or the transformation of oxygen sites to carboxylic sites. The choice of ammonium peroxydisulfate is based on its ability to oxidize the surface without modifying drastically the porous structure and enhance surface acidity by increasing carboxylic group presence^22–24^. To our knowledge, there are no reports on the oxidation of activated carbon from sewage sludge blended with waste coal using ammonium peroxydisulfate. Finally, the oxidized activated carbons are tested for nitrate and MR adsorptive remediation.

**Table 1:** Literature oxidized activated carbon extracted from sewage sludge (SBAC) and adsorption capacity.

References	SBAC state	Modification conditions			S_BET_ (m^2^/g)	pH_PZC_	Adsorption
		Oxidant concentration	Soaking time (h)	Temperature (°C)			
^18^	Unoxidized	–	–	–	1003.8	4.02	Malachite Green: 269,54 mg/g
	Oxidized (2 stages: 1 and 2)	1: 2M HNO_3_	1: 3	1: 60	838.3	3.78	Malachite Green: 303,03 mg/g
		2:-	3	2: 300			
		1: 2M H_2_SO_4_	1: 3	1: 60	960	3.66	Malachite Green: 284,90 mg/g
		2:-	3	2: 300			
^11^	Unoxidized (bagasse/sludge: 1/2)	–	–	–	804.57	–	Pb^2+^:-
	Oxidized (bagasse/sludge: 1/2)	60% HNO_3_	6	90	69.19	–	Pb^2+^: 51.3 mg/g
^16^	Unoxidized	–	–	–	721	–	Fluorene: 0.5 mg/g
	Oxidized	10 M HNO_3_ 1g:10 ml	4	90	86.1	–	Fluorene: 2.8 mg/g
^17^	Unoxidized	-	–	–	721.2	–	Pb^2+^: 1.25 mg/g
	Oxidized	10 M HNO_3_ 1g:4 ml	4	20	674.7	–	Pb^2+^: 3.04 mg/g
		10 M HNO_3_ 1g:10 ml	4	90	86.12	–	Pb^2+^: 4.05 mg/g

## Experimental

### Materials

All reagents, including NaOH, NaNO_3_, KOH, HCl (32%), (NH_4_)_2_S_2_O_8_ (APS), H_2_SO_4_, and NaCl, were obtained from Associate Chemical Enterprise (ACE) and were analytical grade. Sigma-Aldrich (SA) supplied commercial activated charcoal (C3014-500G), which was designated Com-AC.

A waste coal sample was collected (using the ply sampling method) from a coal beneficiation facility located 15 kilometers south-west of Witbank in the South African province of Mpumalanga. The coal sample was dried in the laboratory for 48 h and then blended and divided using a Rotary splitter (Eriez) before being placed in a tight bag. A portion of the coal sample was pulverized to a particle size of 0.150–0.250 mm for further physicochemical tests, while another portion was crushed to a particle size of − 1 mm (80%) for petrographic examination. The physicochemical analyses of the waste coal sample utilized in this study are summarized in the Table 2 below. Sewage sludge reject from wastewater treatment facilities can be designated as primary or secondary sewage sludge based on its origin. The first type is the underflow from settling vessels following inlet physical treatment (sedimentation, flocculation, filtration, etc.) and is predominantly inorganic; the secondary, also known as biological sludge, is generated from biological process (activated sludge).

**Table 2:** Nomenclature and conditions of the activation experiments.

Name	Sludge	Discard coal	Concentration KOH: mol/l	Temperature (°C)
D-3-600	D	No	3	600
D-3-750	D	No	3	750
D-5-600	D	No	5	600
D-5-900	D	No	5	900
D-7-600	D	No	7	600
DC-3-900	D	Yes	3	900
DC-5-600	D	Yes	5	600
DC-5-750	D	Yes	5	750
DC-7-900	D	Yes	7	900
S-3-600	S	No	3	600
S-3-900	S	No	3	900
S-5-600	S	No	5	600
S-7-900	S	No	7	600
SC-3-600	S	No	3	900
SC-3-900	S	Yes	3	900
SC-5-900	S	Yes	5	900
SC-7-600	S	Yes	7	600
SC-7-750	S	Yes	7	750

In this paper we employ two types of sewage sludge collected at the East Rand Water Care Company (ERWAT) wastewater treatment plant facility: D, which was acquired during the dissolved air flotation stage, and S, which was a combination of primary and secondary sludge from the digestion and dewatering stages respectively.

### Methods

Raw sewage sludges were sun dried for 24 h before being oven-dried. The following nomenclature is used throughout this paper to refer to all synthesized adsorbents: the letter C denotes the combination of sludge D or S and discard coal C, i.e. DC or SC, followed by the reagent concentration and finally the pyrolysis temperature. DC-5-750, for example, refers to sewage sludge D combined with waste coal C-The reagent concentration is 5M KOH, and the pyrolysis temperature is 750 °C, as stated in Table 2. The capital letter M denotes that the adsorbent has been changed, while the numbers 1 or 2 indicate the oxidation conditions using 1M or 2M ammonium peroxydisulfate (APS), respectively. SBAC (*(*Sludge Based Activated Carbon) refers to all activated carbons synthesized using sewage sludge alone or in conjunction with waste coal in this study. Dried and crushed precursors (sewage sludge with/without waste coal in a proportion of 0% and 50%) were impregnated with KOH solution with concentrations of 3, 5 and 7 moles/l, and a ratio of activating reagent to the sludge of 1.5:1. After impregnation, the mixture was dried for 24 h at 105 °C. The dried impregnated precursors were crushed to 100% passing through a 150 µm sieve, then placed in a crucible and pyrolyzed in an inert atmosphere for 90 min in a muffle furnace connected to a nitrogen bottle with a flowrate of 50 ml/min. The furnace regulator was turned off when the dwell time was reached, and the pyrolyzed sewage sludge was cooled to room temperature and then washed. The washing stage is divided into two stages: acidic and water. The former was performed using a 3 M HCl solution to remove excess activating reagent and ash-laden soluble ash, whilst the latter was performed using distilled water until the filtrate achieved a neutral pH value of between 6 and 7. SBAC (Sludge Based Activated Carbon) was washed and dried for 24 h at 105 °C, then crushed and sieved to a mesh size of 125 μm before being kept in an airtight container in the desiccator for further testing. The dried sludge was crushed to 100% passing through 125 µm sieves, and pyrolyzed at different temperatures (DC-5-750, DC-7-900, SC-3-600, and SC-5-900). The products from the pyrolysis were mixed with discard coal in a proportion of 1:1 and activated with KOH under N_2_ atmosphere at a flow rate of 5 l/min. To achieve surface modification via oxidation, 1 g of pyrolyzed sewage sludge was incubated in 25 ml of 1M or 2M APS dissolved in 1M H_2_SO_4_ at 60 °C for 240 min before being washed with distilled water until the pH neutral value was reached. Batch adsorption experiments were carried out with an adsorbent dose of 0.5% (5 mg of adsorbent in 10 ml of solution) and the performance of adsorbents was compared to commercial activated carbon. The best oxidized and/or unoxidized SBACs were chosen based on preliminary evaluation test results conducted at 303 K in an incubator shaker. The initial concentration of nitrate was 50 mg/l, with contact times of 180 and 360 min, respectively, while the initial concentration of MR was 75 ppm, with contact times of 90 and 180 min, and initial pH of 2 and 4 for nitrate and methyl red, respectively. The initial pH value of 2 in the case of nitrate was chosen because adsorbent surfaces are prone to being positively charged as pH decreases, and nitrate ions cannot compete with hydroxyl anions^25^ and pH 4 was chosen in the case of MR to prevent competition with H^+^ cations while attempting to preserve the adsorbent surface deprotonated^26^.

### Chemical analysis

SEM and EDS analysis were done using a ZEISS SIGMA FESEM 03- 39 apparatus on coated samples on a carbon tape support using Gold-Palladium techniques. TGA was performed with the Pioneer (SDT-Q500) equipment. Proximate analysis of sewage was performed using a Perkin-Elmer STA 6000 simultaneous thermal analyser. FT-IR analyses were done using the Perkin-Elmer FT-IR Spectrometer Spectrum 2. Ultimate analysis was conducted with the thermoscientific flash 2000. XRD study was done using a Bruker 2D phaser instrument. The NH_4_OAC method^27^ was used to determine cations exchange capacity. The Boehm titration method was used to determine the acidity of SBAC^28^. The pH point of zero charge was determined using the procedure described by Leng et al.^29^. Surface functionalities and graphitization or carbon disorder structure of the adsorbent were assessed using FT-IR analysis and Raman spectroscopy, respectively. For the pH point of zero charge, 50 mg of adsorbent were placed in vials with 40ml of 0.01 M NaCl solution and shaken for 48 h, the solution pH ranged from 2 to 12 with an incremental of 2. The concentrations of nitrate and MR were calculated using an IC dionex-120 and a U-vis Shimadzu 1800, the latter at a wavelength of 480 nm, as per Ding et al.^30^.

## Results and discussion

### Preparation and characteristics of precursors

The precursors were sun dried, and the moisture content of the sample was determined by mass losses after oven drying at 105 °C for 24 h as shown in Table 3.

**Table 3:** Non-dried and sun dried sample moisture content.

Types of sludge	Non-dried sample moisture (%)	Moisture content after sun drying (%)
D	96.28	6.285
S	85.33	7.037

The elemental analysis (Table 4) reveals that there is only a minor difference between the two samples, indicating that digestion has no effect on the organic content of sewage sludge, despite the volatile solid content being slightly different. However, although the surface area of sludge from D was approximately 1.5 m^2^/g, the surface area of sludge S could not be determined, possibly due to a lack of porosity in the precursor materials.

**Table 4:** Precursors properties.

	Precursors		
	D	S	Discard coal
**Elemental analysis**			
C (%)	36.511	36.640	43.133
H (%)	5.526	5.215	2.424
N (%)	5.020	2.681	0
S (%)	2.002	2.225	2.429
H/C	0.151	0.142	0.056
N/C	0.137	0.073	0
**Proximate analysis**			
Fixed carbon (%)	4.15	4.86	15.76
Ash (%)	29.15	35.98	67.96
Volatile (%)	66.7	59.16	16.28
Volatile solid	2.161	1.216	1.474
**Surface properties**			
S_BET_ (m^2^/g)	1.411	–	3.848
Pore volume	0.011	–	0.014
Pore size (nm)	30.72	–	15.11

**Figure 1:**
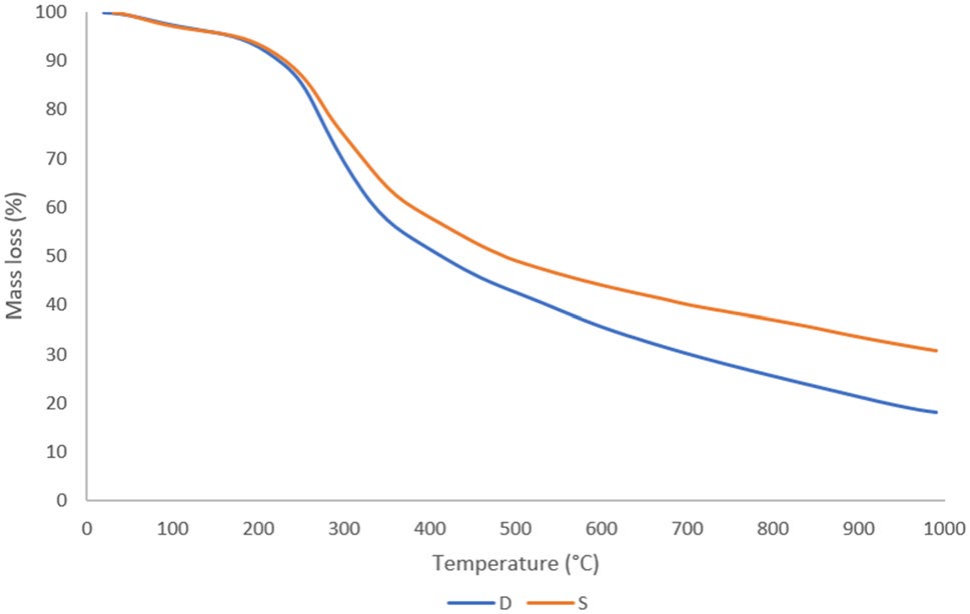
Thermogravimetric curves of raw sewage sludge D & S.

In Figure 1, the TGA curve of dried sludge shows that between 30 and 190 °C, there is a slight mass loss, and the mass of both sludge was roughly 5%, possibly due to adsorbed water. The most mass loss occurred between 200 and 600 °C, with mass loss of D and S increasing from about 5% at 200 °C, to about two-thirds (64%) and half (46%) at 600 °C respectively. The mass loss from 600 to 1000 °C is less pronounced for both sludge with 17 and 13% for D and S respectively. As a result, 600 °C was chosen as the minimum temperature for pyrolysis. S appears to be slightly more stable than D based on the TGA curves.

**Figure 2:**
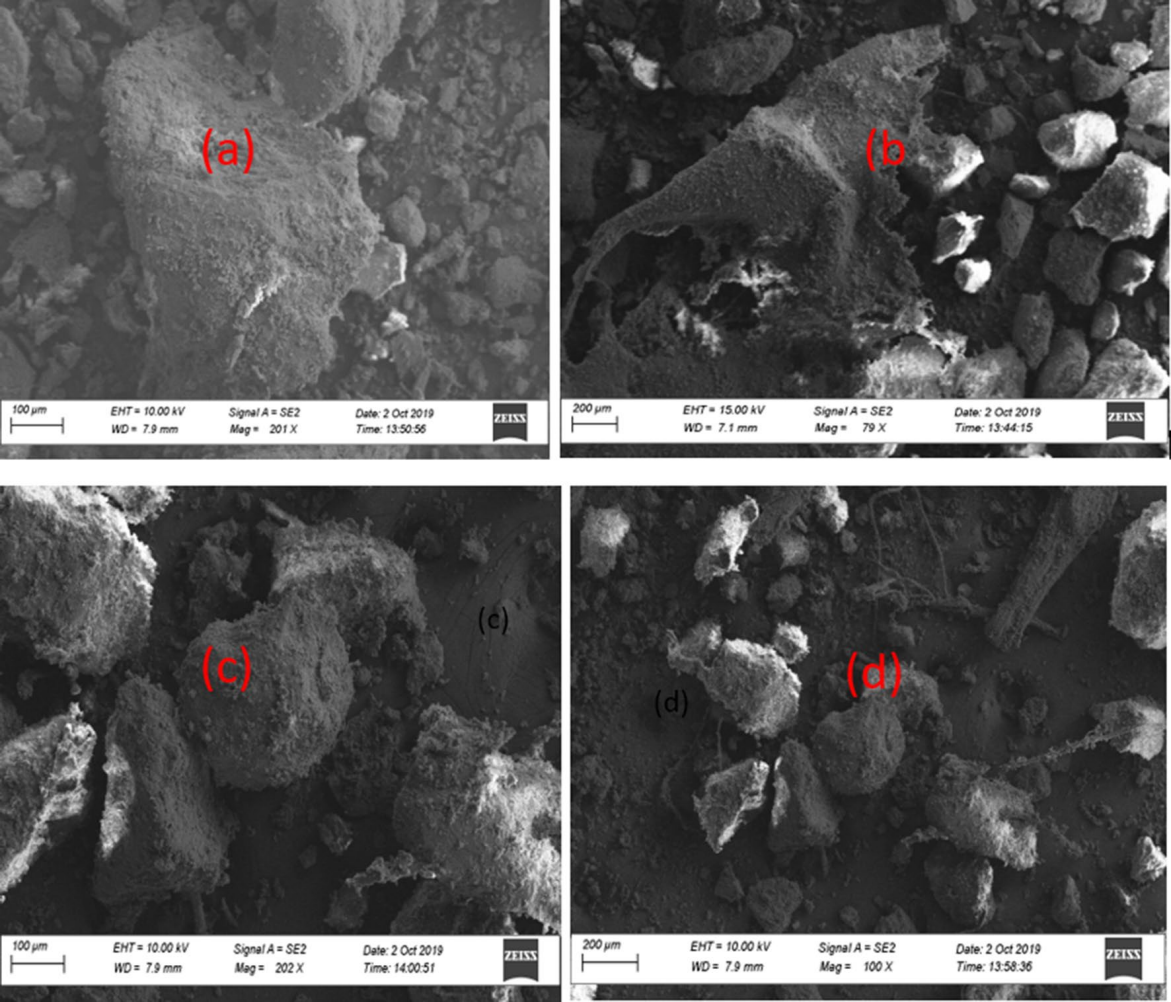
microscopic observation of raw sewage sludge D: (**a**) and (**b**), S: (**c**) and (**d**).

**Figure 3:**
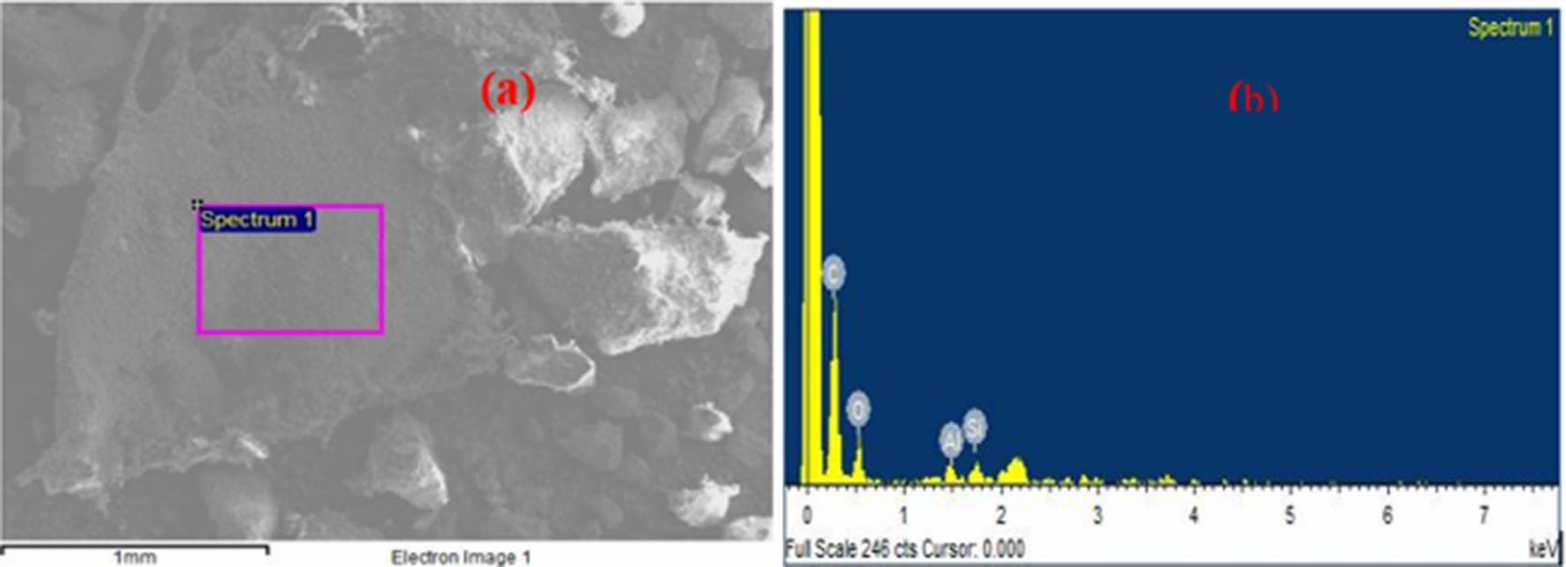
Elemental analysis of raw sewage sludge D: (**a**) and (**b**).

**Figure 4:**
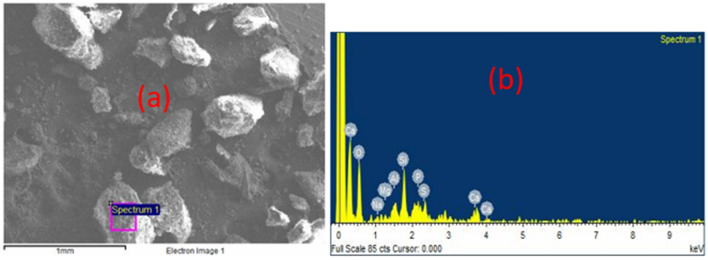
Elemental EDS analysis of raw sewage sludge S: (**a**) and (**b**).

Both samples D and S show heterogeneous morphology and composition as revealed by microscopic examination by SEM analysis in Fig. 2a–d, and EDS analysis in Figs. 3a, b and 4a, b respectively. The sponge-like morphology shown in Fig. 2b may be attributed to an organic water-repellent compound that adhered to the bubbles during the dissolved air flotation process. Both Figs. 3a, b and 4a, b reveal that the main elements in the sewage sludge samples were carbon and oxygen, with silica and aluminium at trace concentrations. In comparison to D sludge, energy dispersive X-ray (EDX) analysis of sample S indicated a high inorganic content, which was most likely created by domestic wastewater particles recovered during decantation or solid-liquid separation. This can be explained by the fact that excess air is released during the dissolved air flotation (DAF) process in the form of microbubbles that adhere to the dispersed phase, causing the particles to float, resulting in the formation of aerophobic compounds that are predominantly inorganic, whereas digestion reduces the amount of organic compounds, most likely due to the production of biogas.

**Figure 5:**
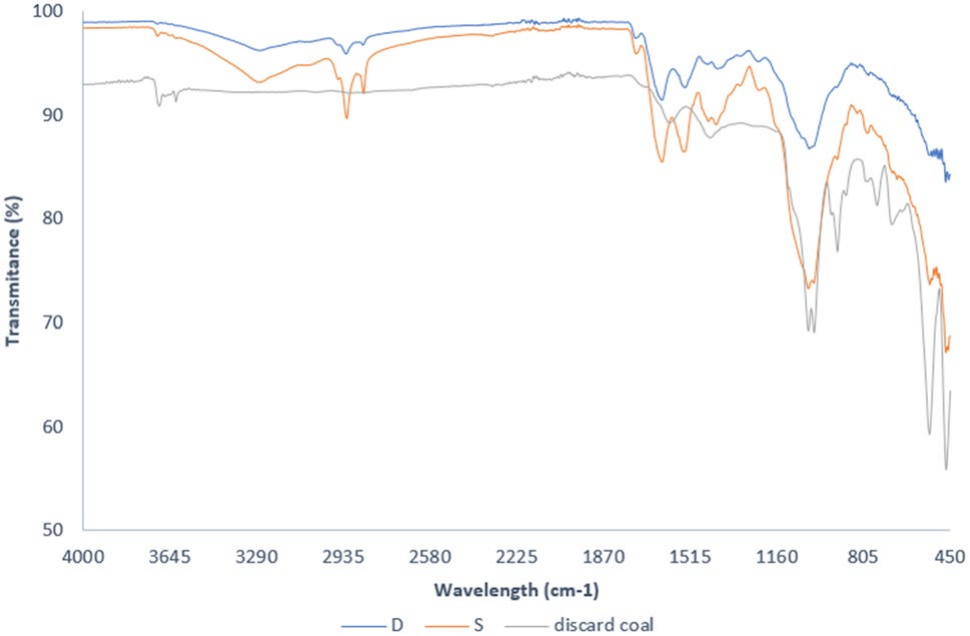
FT-IR spectra for precursors D &S.

Figure 5 shows the FT-IR spectra of the raw sewage sludge with wavenumbers ranging from 450 to 4000 cm^−1^. Reading the peaks spectra wavelengths from left to right, the peak observed at 3688–3619 cm^−1^ is attributed to OH–kaolinite and gibbsite lattice stretching^31,32^, 2988–2901 cm^−1^ to –C–H group vibration^7,33^, 1631 cm^−1^ and 1538 cm^−1^ to sulphur and nitrogen functional groups, respectively^33^. The shape of the shoulder peaks at 1050–1090 cm^−1^ was attributed to Si-C or Si-O-Si bands (Liang et al.^15^), C–O–C vibration^33^, and finally, under 1000 cm^−1^, the peaks at 749 cm^−1^, 535 cm^−1^, and 467 cm^−1^ were attributed to silica or calcium carbonate stretching^32,34^. Discard coal has a lower transmittance from 1498 cm^−1^ peaks than FT-IR spectra sludge 1007 cm^−1^, 1030 cm^−1^, implying that waste coal contains more mineral elements whereas sludge S has lower transmittance than D, implying that the former contains more functional classes.

### Characteristics of synthesized activated carbons

**Figure 6:**
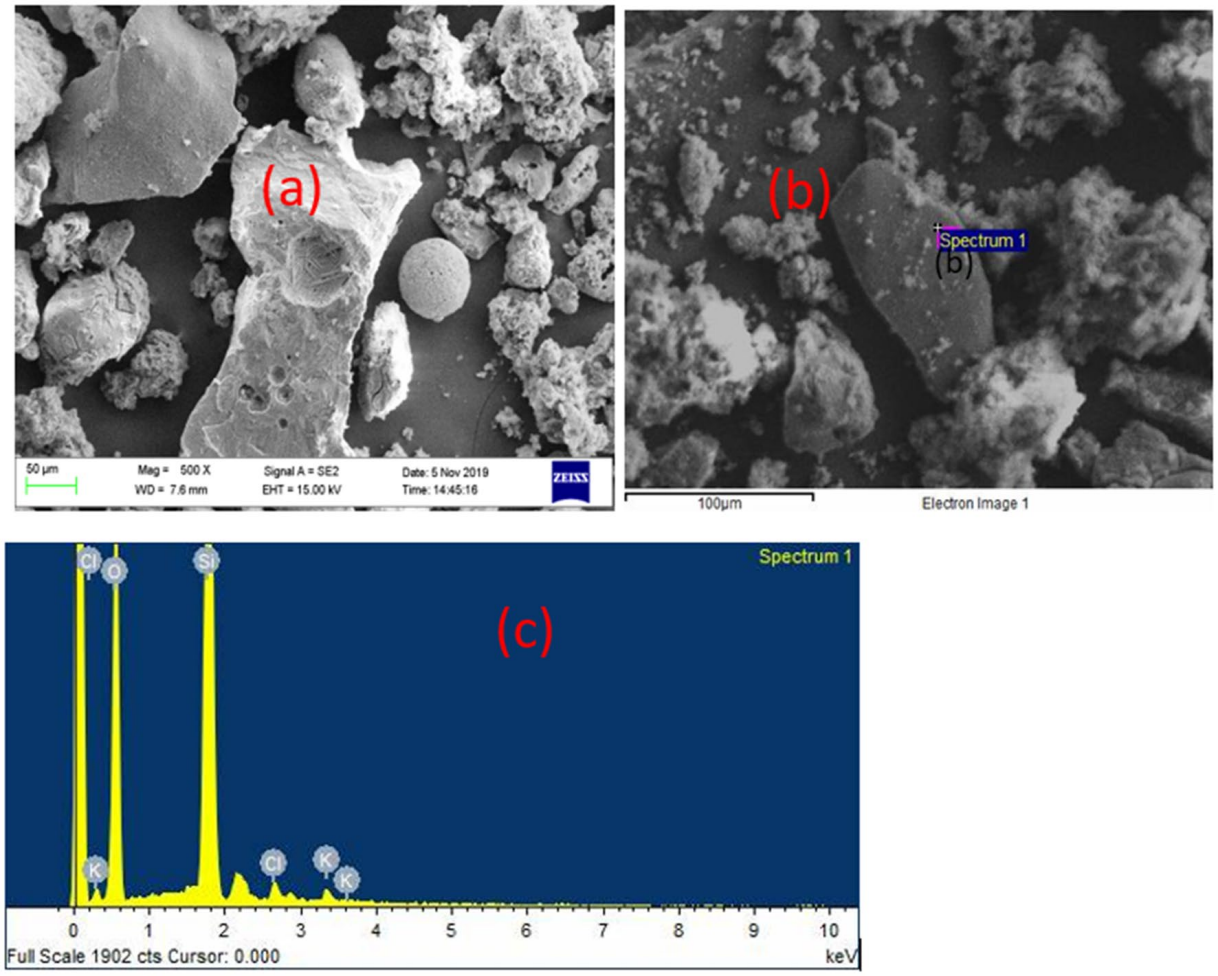
SEM microscopic analysis of SBAC SC-3-600, (**a**) multiple particle (**b**) focus on particle without cavity after activation and (**c**) qualitative analysis of the targeted particle.

Figure 6.a shows the microscopic examination of the sorbent SC-3-600 after activation. Pyrolysis and washing stages may have facilitated cavity formation of highly formed cavities due to the remarkable depletion of inorganic and organic components^35^. Furthermore, some particles (Fig. 6a, b) lack or have insignificant cavities, which may be attributable to a lack of volatile and decomposed matter escaping to facilitate porosity^9^. The EDS qualitative analysis (Fig. 6c) of the local particle (Fig. 6b) revealed inorganic characteristics as well as the presence of K and Cl from activation and washing, respectively.

**Figure 7:**
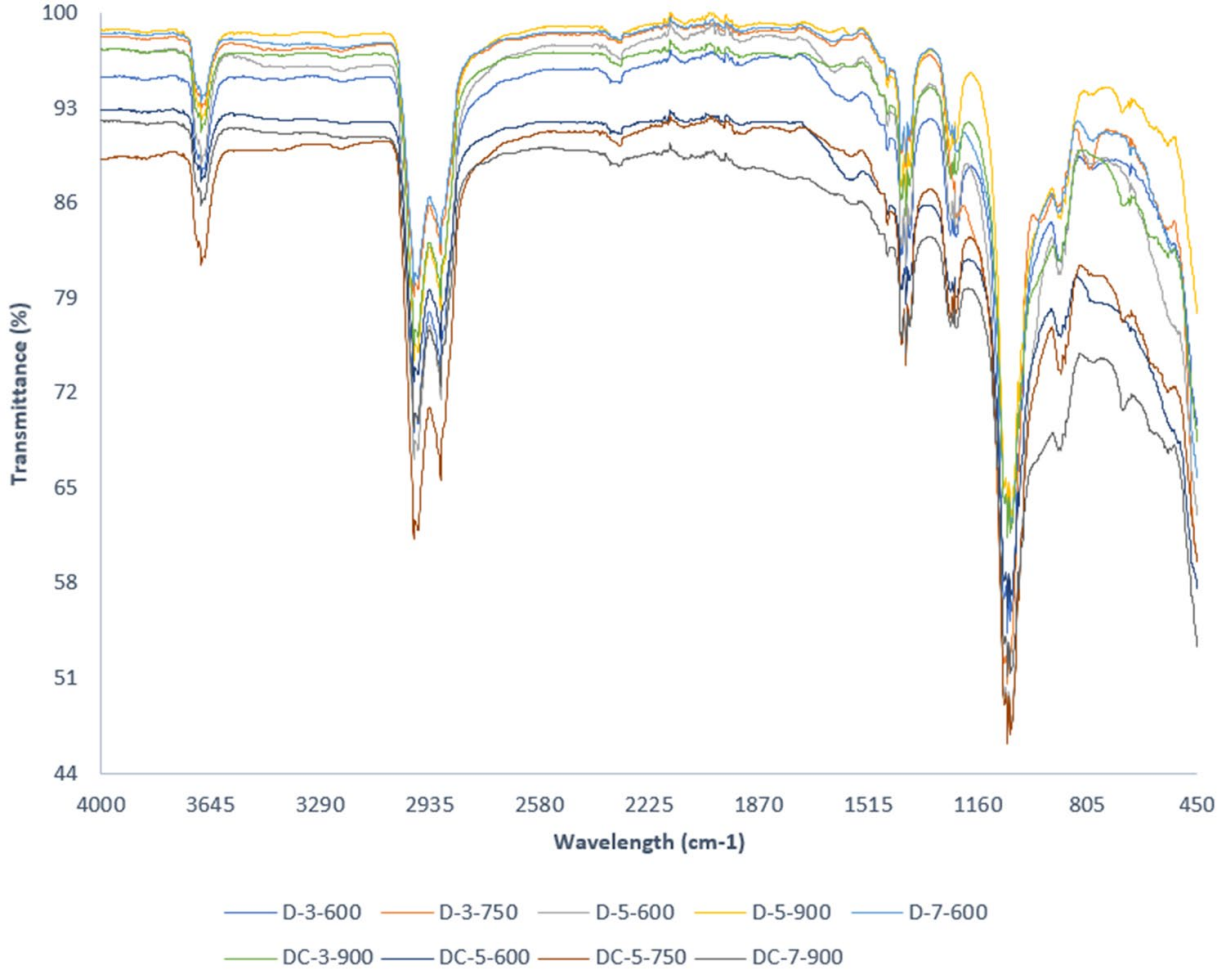
FT-IR spectra of SBAC obtained from raw sewage sludge D as precursor.

**Figure 8:**
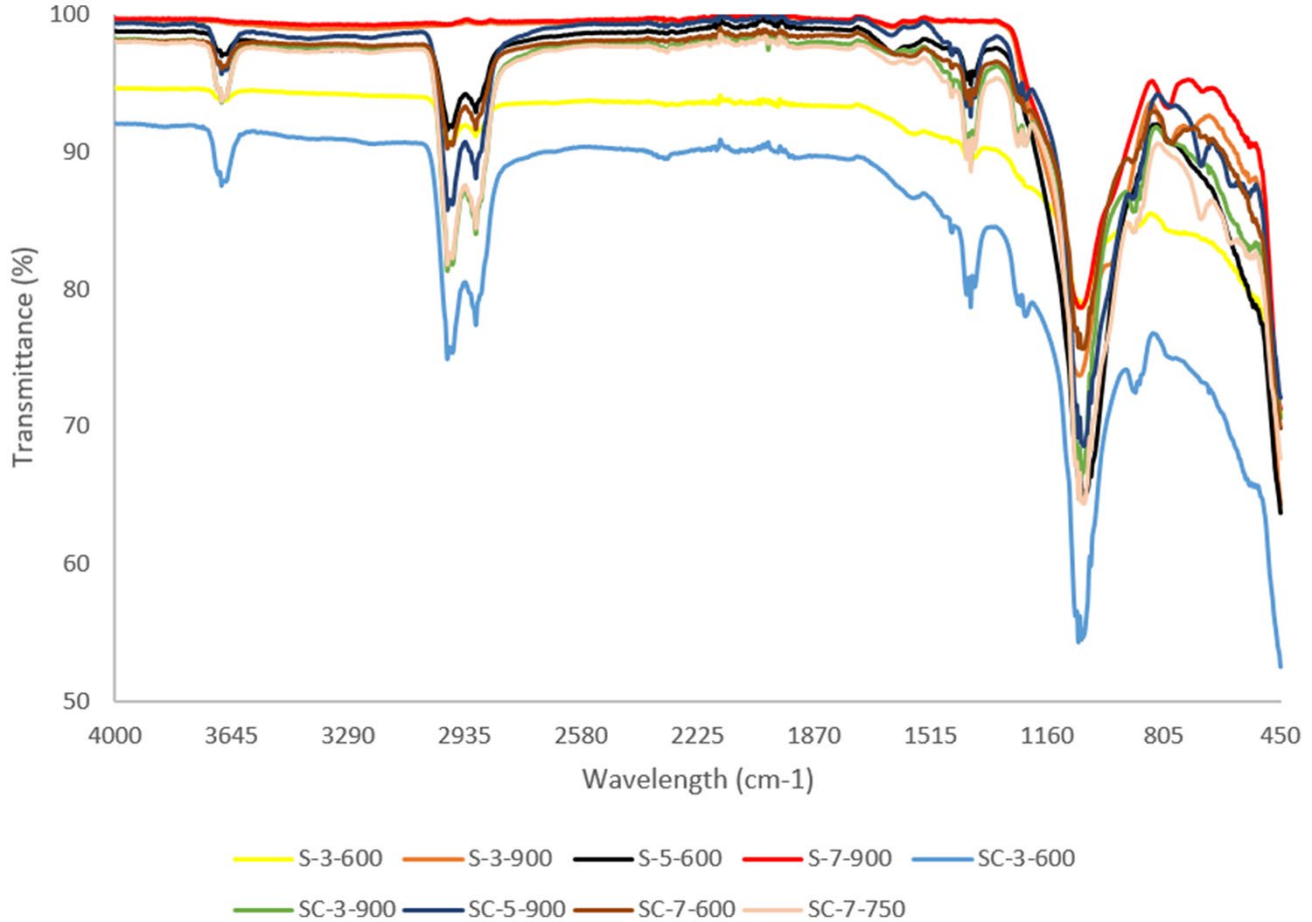
FT-IR spectra of SBAC obtained from raw sewage sludge S as precursor.

The FT-IR spectrum of the various SBAC (as per Table 4) exhibited almost identical shape and peaks with different intensities regardless of the parameter involved in the synthesis process, as shown in Figs. 7 and 8. The spectra have six main peaks, which are 3676 cm^−1^, 2901–2998 cm^−1^, 1394 cm^−1^, 1225 cm^−1^, and 892 cm^−1^ in all SBAC samples.

In comparison to feedstock spectra, the disappearance of the wavelength peak at 470 cm^−−1^ and 500 cm^−1^ in SBAC could be due to inorganic matter solubilisation during the acid washing phase^36–38^, while 798 cm^−1^ could be due to dehydrogenation reactions^24^, 1631 cm^−1^ and 1538 cm^−1^ could be due to thermal degradation of protein for nitrogen related compound or sulphur^33^.

The peaks associated with C-H group stretching not only shifted slightly from2859 to 2922 cm^−1^ in feedstock to a higher value (2901–2988 cm^−1^) in SBAC, which can be related to the presence of saturated group^11^ but also transmittance increased after activation for all samples, which is in contrast to some literature^7,33,39^ in which it was argued that the disappearance of the peaks was due to the the decomposition of fatty organic matter and dehydration^7^. Organometallic formation may be a possible explanation for the increased transmittance of SBAC pyrolyzed at lower temperatures followed by depletion as temperature rises; for example, the abundance of functional groups (hydroxyl, carboxyl) on the biochar surface synthesized from sewage sludge pyrolyzed at 300 °C reduced extractable cations due to the formation of organometallic compounds^40,41^. The functional groups present in SBAC can be summarized as O–H, C=C, C=O, aliphatic C–H, Si–C, Si–O–Si, phosphate, and carbonate, based on the observations.

**Figure 9:**
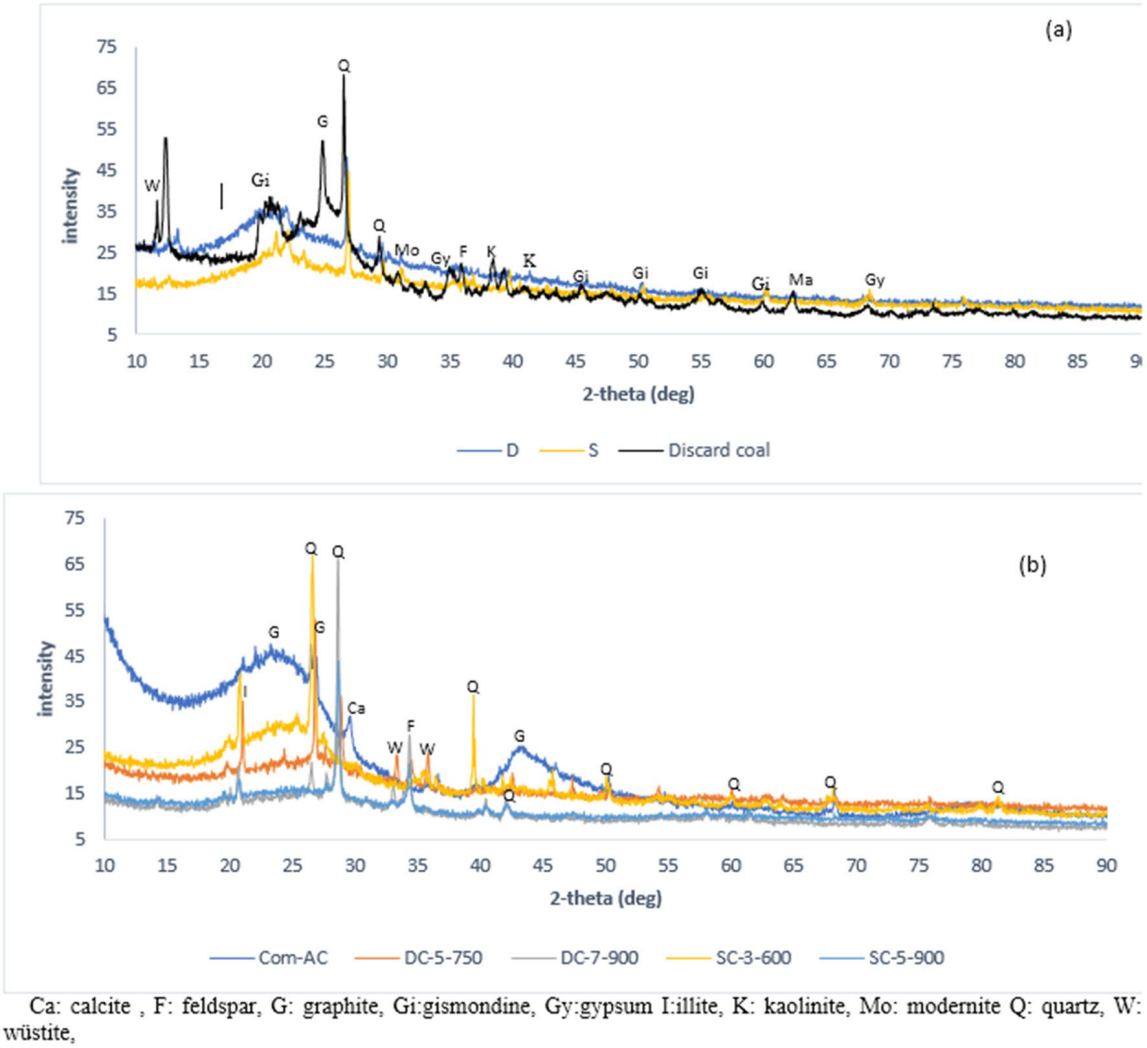
XRD graph of feedstock (**a**), SBAC and Com-AC (**b**).

Com-AC has broad peaks (2 = 25.3° and 2 = 44.6°) linked to its amorphous phases, while the XRD pattern (Fig. 9a, b) demonstrated mineral phases transformation from broad peak in the precursors to sharp in the manufactured absorbent, which clarified transition from amorphous to crystalline phase due to pyrolysis. XRD verified the existence of minerals such as wustite, quarts, illite, and feldspars in SBAC. It is worth noting that the presence of alkaline earth elements in the minerals (feldspars and illite) led to magnesium, calcium, and iron being classified as exchangeable cations.

### Surface modification of activated carbons through oxidation

**Figure 10:**
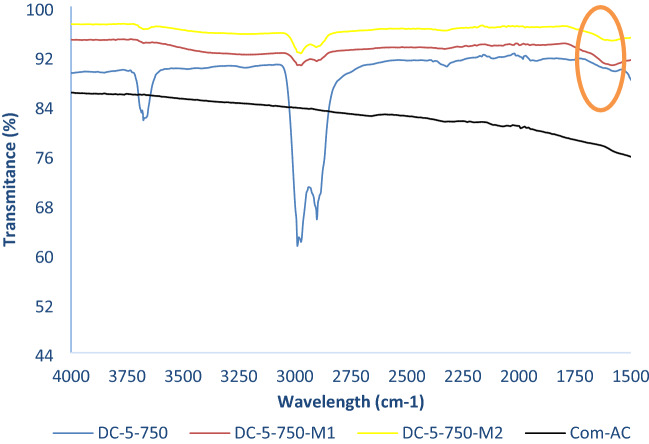
FT-IR spectra of Com-AC, DC-5-750 and its two derived oxidized sorbents.

The oxidation of SBAC resulted in surface functionalities modifications with formation of peaks in the oxidized adsorbent around 1550–1575 cm^−1^ (Fig. 10), which can be linked to symmetric COO– and nanoaromatic C=O stretching entailed in formation of carboxylic functional groups or C=C bond of the aromatic skeleton ring of adsorbent^13^. On further notice the reduction in –CH (2901–988 cm^−1^) and –OH (3640 cm^−1^) stretching substances which may be ascribed to their solubility/affinity with H_2_SO_4_. On the other side, Com-AC did not have functional group.

**Figure 11:**
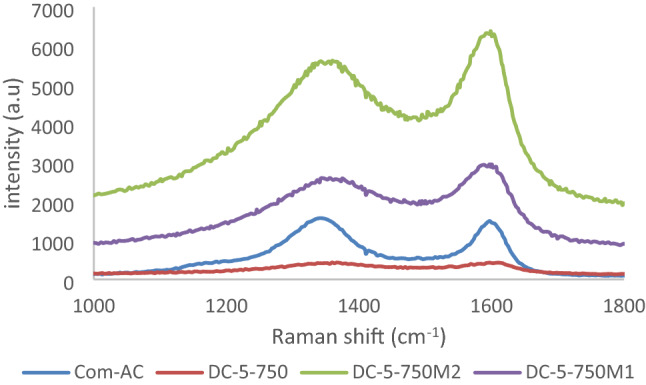
Raman spectra of Com-AC, DC-5-750-oxidized adsorbents.

Raman spectra pattern is shown in Fig. 11, The peak located at 1587cm^−1^ (G band) and 1357 cm^−1^ (D band) are associated to sp^2^-bonded carbon and disorder of carbon structure respectively and their intensity I_G_ and I_D_ reveal the adsorbent degree of graphitization and carbon disorder structure from which I_D_/I_G_ ratio enunciates prevalence of carbon disorder structure over graphitization and vice-versa^13^.

As shown in Table 5, graphitic structure is more predominant than carbon disorder structure and augmented with oxidation, partial graphitization of activated carbon was reported with acidic oxidation treatment with HNO_3_^42^.

**Table 5:** Intensity ratio of peak adsorbents.

Adsorbents	I_D_ (1387 cm^−1^)	I_G_ (1587 cm^−1^)	I_D_/I_G_
Com-AC	1028.126	1397.75	0.735
DC-5-750	385.270	398.887	0.965
DC-55-750M1	2476.854	2921.75	0.847
DC-5-750M2	5262.40	6284.252	0.837

**Figure 12:**
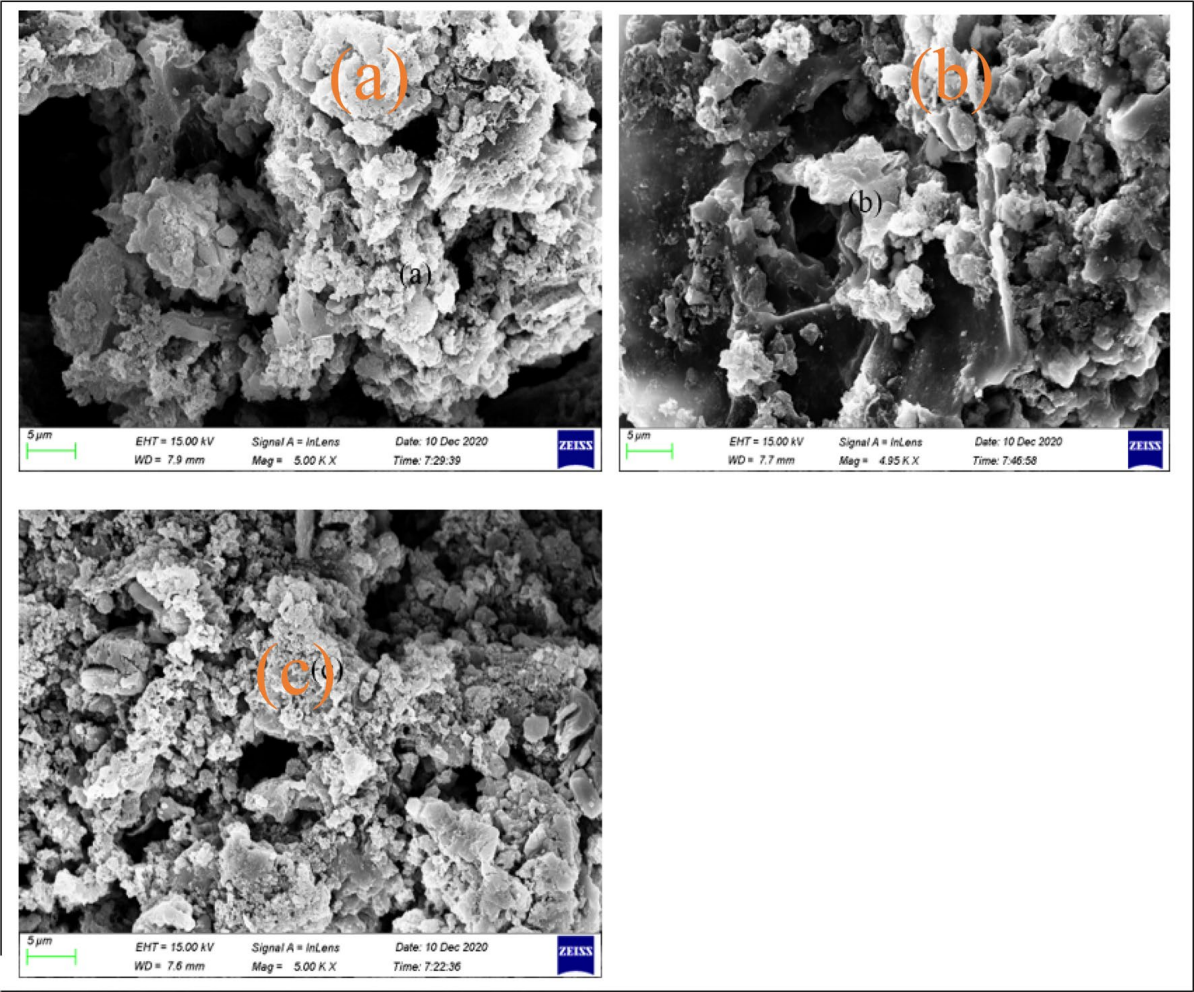
morphology of (**a**) DC-5-750, (**b**) DC-5-750 M1 and (**c**) Com-AC.

**Figure 13:**
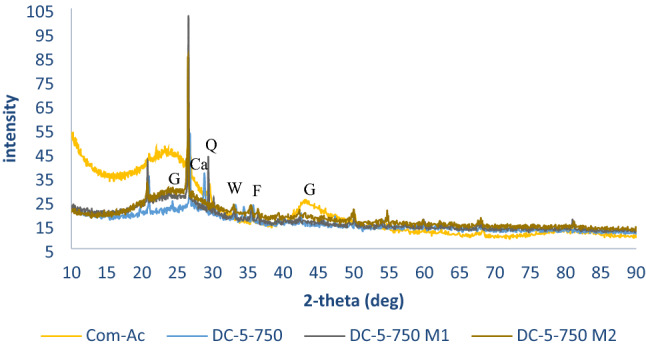
XRD spectra of Com-AC, unoxidized (DC-5-750) and its oxidized sorbent.

The surface of oxidized adsorbent (Fig. 12b) exhibited less irregularities and soften surfaces than the unoxidized adsorbent (Fig. 12a) and Com-AC (Fig. 12c) probably as results of corrosive H_2_SO_4_-adsorbent surface interaction^18^ and also disintegration of pore structure situated at the carbon edge^17^. XRD spectra of adsorbent are represented in Fig. 13 after oxidation treatment the unoxidized sorbent exhibited a broader peak (2θ = 25.3°) associated to amorphous or graphite structure as endorsed by Raman spectra analysis, on the other hand augmentation in peak (2θ = 30°) ascribed to peak might be corollaries of other mineral (feldspar and illite) solubilisation/decline.

The oxidation with APS did not impact significantly carbon composition as per ultimate analysis results since the biggest difference was 6.771% (from DC-5-750: 23,612% to DC-5-750M2:30,383%) while with other sorbents (SC-3-600,SC-5-900,DC-7-900) variation was less than 2%, alike tendency was reported by Ang et al.^22^

Furthermore, textural properties in Table 6 receded severely after modification, in case of adsorbent derived from sludge S, for instance prior oxidation SC-3-600 had surface area of 281.72 m^2^/g that shrunk to 68.22 m^2^/g and 46.673 m^2^/g when treated with a solution of 1M and 2M APS respectively, probably due to thinness of walls pores, which are prone to collapse^43^ and/or micropore occlusion^44^. In the case of DC-5-750, surface area varied slightly (7.28-30.57 m^2^/g: variation) and micropore areas changed from 124.14 to about 157.36 m^2^/g and 176.11 m^2^/g after modification with 1M APS and 2M APS respectively. After modifications DC-7-900 surface area (247.57 m^2^/g) increased probably as results of micropore and mesopore collapse emerging from activated carbon surface etching by reagents, it was 285.59 m^2^/g for DC-7-900M1 and 295,31 m^2^/g for DC-7-900M2.

**Table 6:** Structural properties and ultimate analysis results of SBAC after and before oxidation.

Adsorbent	Modification conditions	S_BET_ (m^2^/g)	S_meso_ (m^2^/g)	S_micro_ (m^2^/g)	V_tot_ (cm^3^/g)	V_micro_ (cm^3^/g)	Pore size (nm)	C (%)	H (%)
Com-AC	–	609.209	195.74	336.236	0.469	0.165	3.14	78.411	0.000
SC-3-600	Unoxidized	281.72	90.942	123.49	0.342	0.075	4.857	45.227	0.7411
	1M APS	68.227	24.098	35.241	0.0953	0.0175	5.58	47.411	1.007
	2M APS	46.573	19.631	14.807	0.085	0.0073	9.02	46.394	1.017
SC-5-900	Unoxidized	422.09	222.394	111.15	0.453	0.0495	4.296	2.014	0.000
	2M APS	313.05	61.156	223.75	0.291	0.117	3.604	1.891	0.000
DC-5-750	Unoxidized	312.79	100.212	124.14	0.366	0.0898	4.681	23.612	0.454
	1M APS	305.51	115.232	157.36	0.421	0.0741	4.51	29.901	1.087
	2M APS	282.22	77.816	176.113	0.299	0.083	4.244	30.383	1.019
DC-7-900	Unoxidized	247.57	129.83	175.60	0.265	0.0320	4.28	15.460	0.000
	1M APS	285.59	117.275	133.148	0.302	0.0666	4.099	14.831	0.0075
	2M APS	295.31	103.47	121.553	0.289	0.061	3.923	15.111	0.0079

### Adsorption kinetics

To better understand kinetics order and intra particle diffusion, the effect of time was investigated using the following relationships:

1 $$ q_{e} = \frac{{\left( {C_{0} - C_{e} } \right) \times V}}{m} $$

2 $$ Rp = \frac{{\left( {C_{0} - C_{e} } \right)}}{{C_{0} }} \times 100 $$

where q_e_ (mg/g), C_0_ (mg/l), C_e_ (mg/l), m (mg) and V (ml) represent the adsorption capacity, initial concentration, equilibrium concentration, adsorbent mass and volume of liquid in contact with adsorbent respectively. The removal (%) is calculated via Eq (2).

The first and second order kinetics models, as well as interparticle diffusion, are commonly used to understand the adsorption mechanism of pollutants with activated carbon^45^.

Adsorption kinetics can be expressed in terms of the hypothesis that adsorbate removals obey a first-order kinetics:

3 $$ \frac{dq}{{dt}} = K_{1} \left( {q_{e} - q_{t} } \right) $$

where q_t_ and q_e_ are the amount of pollutants adsorbed per mass of adsorbent (mg/g) at the targeted time and equilibrium, respectively, and k_1_ is the constant rate (min^−1^).

After integration with conditions that q_e_ = 0 if t = 0, Eq (3) can be written:

4 $$ ln\left( {\frac{{q_{e} - q_{t} }}{{q_{e} }}} \right) = - K_{1} t $$

Or alternatively

5 $$ ln\left( {1 - \frac{{q_{t} }}{qe}} \right) = ln q_{e} - K_{1} t $$

The adsorption capacity at equilibrium (q_e_) and the first-order sorption rate constant (*K*_1_) can be evaluated from the slope and the intercept respectively from plot of ln (1 − q_t_/q_e_) vs t.

The second pseudo order kinetics is defined by equation:

6 $$ \frac{dq}{{dt}} = K_{2 } \left( {q_{e} - q_{t} } \right)^{2} $$

where *K*_2_ is the second order rate constant, integration of Eq (6) with initial conditions when t = 0 and q_e_ =0, lead to:

7 $$ \frac{t}{{q_{t} }} = \frac{1}{{K_{2} q_{e}^{2} }} + \frac{t}{{q_{e} }} $$

*K*_2_ and q_e_ can deducted from the slope and the intercept of the plot t/q_t_ vs t, where q_t_ is the adsorption capacity at a specific time.

The intraparticle diffusion model is a convenient means to depict diffusion mechanism and examine whether intraparticle diffusion is the rate-limiting step in the adsorption process. The intraparticle model diffusion is represented by:

8 $$ q_{t} = K_{int} t^{1/2} + C $$

*K*_int_ and C are determined from the slope and intercept of q_t_ vs t^1/2^.where *K*_*in*t_ is the intraparticle diffusion rate constant (mg g^−1^ min^−1/2^) and C is the boundary layer effect intercept; the larger C, the greater the contribution of surface sorption to the rate-controlling step.

**Figure 14:**
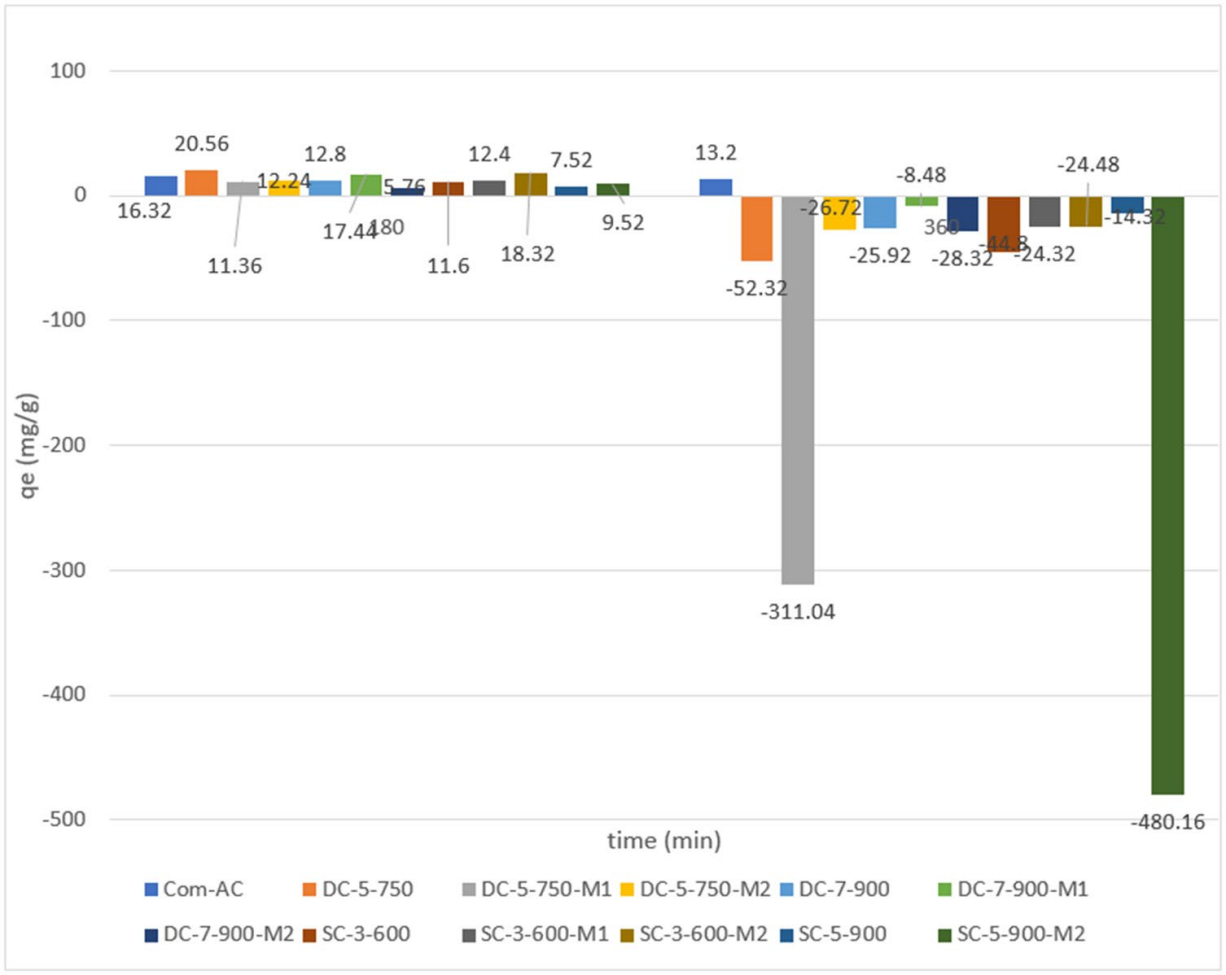
preliminary adsorption capacity for nitrate.

The “preferred” oxidized and unoxidized SBAC were chosen based on removal evaluation tests performed at a constant temperature of 303 K for 180 and 360 min with an initial concentration of 50 mg/l of nitrate, 90 min and 180 min for MR with 75 ppm as an initial concentration and an initial pH of 2 for nitrate and 4 for methyl red. The initial pH of 2 in the case of nitrate was chosen based on the hypothesis that adsorbent surfaces become positively charged as pH decreases, preventing competition with hydroxyl anions and pH 4 in the case of methyl red to avoid competition with H^+^ cations while attempting to keep the surface deprotonated (below the pH_pzc_ for the oxidized adsorbent) as MR pKa = 5.1. Initially, unoxidized SBAC outperformed oxidized SBAC as shown in Fig. 14, probably due to the former's proclivity for having a highly deprotonated surface at lower pH. The adsorption capacity decreased as time progressed, with the exception of Com-AC, where it changed slightly from 16.32 to 13.2 mg/g for contact times of 180 and 360 min, respectively. Furthermore, SBAC (DC-5-750M1 and SC-5-900M2) released a significant amount of nitrate ion in solution after 360 min of agitation, while this amount was lower when the process was carried out with other adsorbents. This situation may be related to the release of compound contained in the adsorbent ash in the liquid phase and/or the adsorption equilibrium phenomenon, in which optimum equilibrium contact was reached. DC-5-750 and SC-3-600, as well as the oxidized sorbents DC-5-750M2 and SC-3-600M2, were chosen for further nitrate adsorption experiments. The adsorbent had mesopores, making it ideal for removing medium-sized substances from liquid process^1^. Despite having a larger surface area, SC-5-900 (422.09 m^2^/g) and SC-5-900M2 (313.05 m^2^/g) had a lower adsorption capacity than other SBAC. This may be attributed to lower carbon content (Table 1) or the disappearance of acidic functional groups as temperatures rose^36^, and this result confirmed the significance of functional group presence. Based on the test results in Fig. 6, DC-5-750 M1 and DC-7-900 M1, with adsorption capacities of 127.634 mg/g and 124.376 mg/g, respectively, were chosen for additional experiments in relation to Com-AC (121.10 mg/g) performances, as well as unoxidized SBAC (DC-5-750 and DC-7-900). Figure 7a depicts the nitrate removal pattern over time. Prior to 30 min of contact time, all adsorbents had negligible adsorption power, most likely due to film diffusion resistance (external diffusion)^46^, but it increased significantly up to 120 min for Com-AC, DC-5-750, and DC-5-750M1 at 15.132 mg/g, 17.46 mg/g, and 10.72 mg/g, respectively.

**Figure 15:**
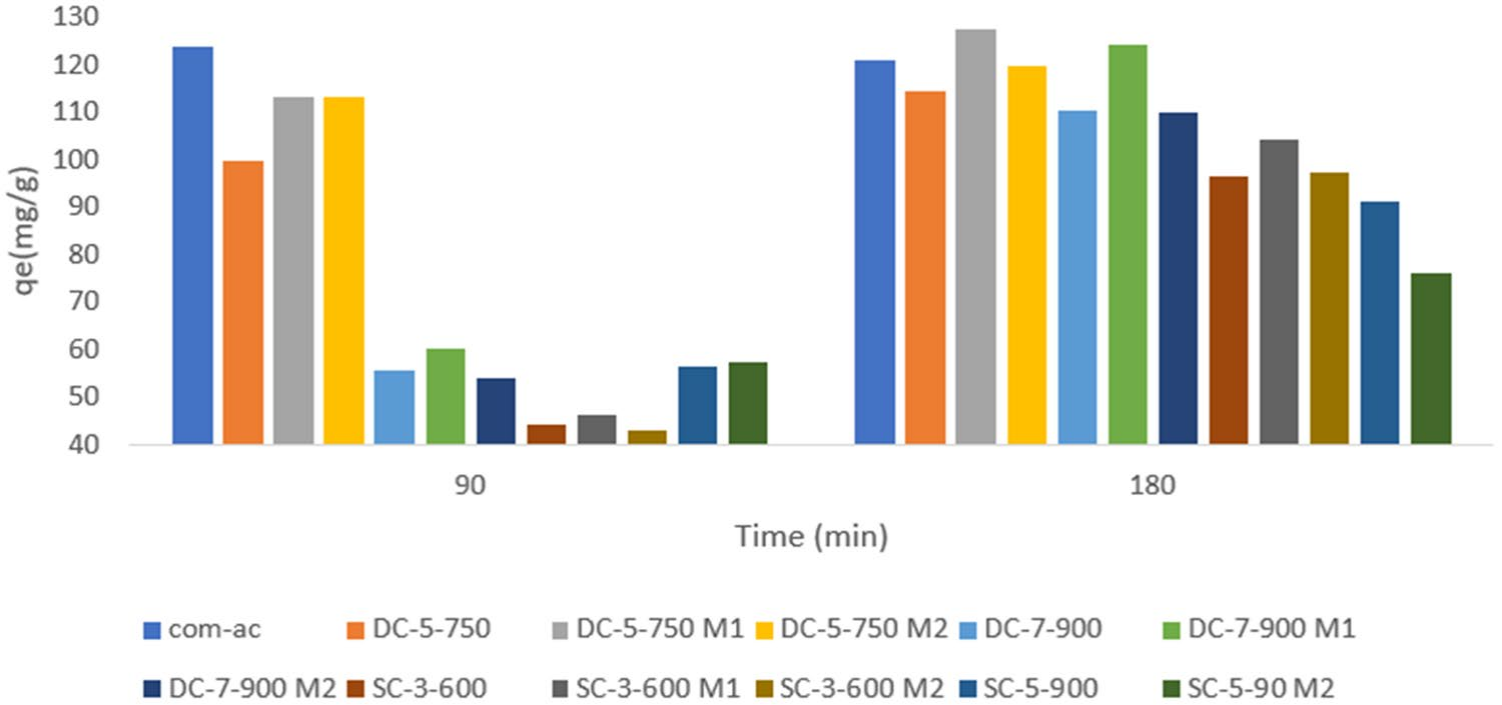
preliminary adsorption capacity results for MR.

As illustrated in Fig. 15, adsorption rises with time except for Com-AC, where the change was insignificant (from 123. 8 mg/g at 90 min to 121. 2 mg/g at 180 min), owing to the fact that equilibrium had already been established. Despite having the highest surface area, SC-5-900 and SC-5-900M2 had a lower q_e_ than other sorbents, most likely due to a lower carbon content (Table 6) or a lack of acidic functional groups due to their depletion during temperature augmentation^14^. This finding emphasizes the critical nature of functional group presence.

As depicted in Figure 15, the synthesised sorbents with the highest adsorption potential were DC-5-750 M1 (127.6 mg/g) and DC-7-900 M1 (124.4 mg/g), and they were therefore chosen for future experiments, along with the unmodified sorbents (DC-5-750 and DC-7-900).The upward tendency may be associated to pore diffusion and surface reaction which are deemed to present less component resistance than external diffusion^46^, while stagnant trend after 120 min may be ascribed to occupation of available adsorption site by nitrate ions as the process progress^25,47,48^, which weaken the interaction between sorbate and adsorbent surface^48^.

Preliminary tests shown in Fig. 6 revealed that the adsorption capacity increased significantly with time with a little fluctuation in Com-AC from 123.8 mg/g at 90 min to 121.172 mg/g at 180 min, implying that equilibrium had already been reached.

Although having a higher surface area, the adsorption capacity of SC-5-900 (422.1 m^2^/g) and SC-5-900M2 (313.1 m^2^/g) was lower than other SBAC. This could be due to lower carbon content (Table 5) and/or depletion of acidic functional groups as they vanished with temperature rise (28). This observation further corroborated the importance of functional group presence.

Based on results in Fig. 6, DC-5-750 M1 and DC-7-900 M1 with adsorption capacities of 127.6 mg/g and 124.4 mg/g, respectively, were chosen for additional investigations in comparison to Com-Ac (121.1 mg/g) performances.

**Figure 16:**
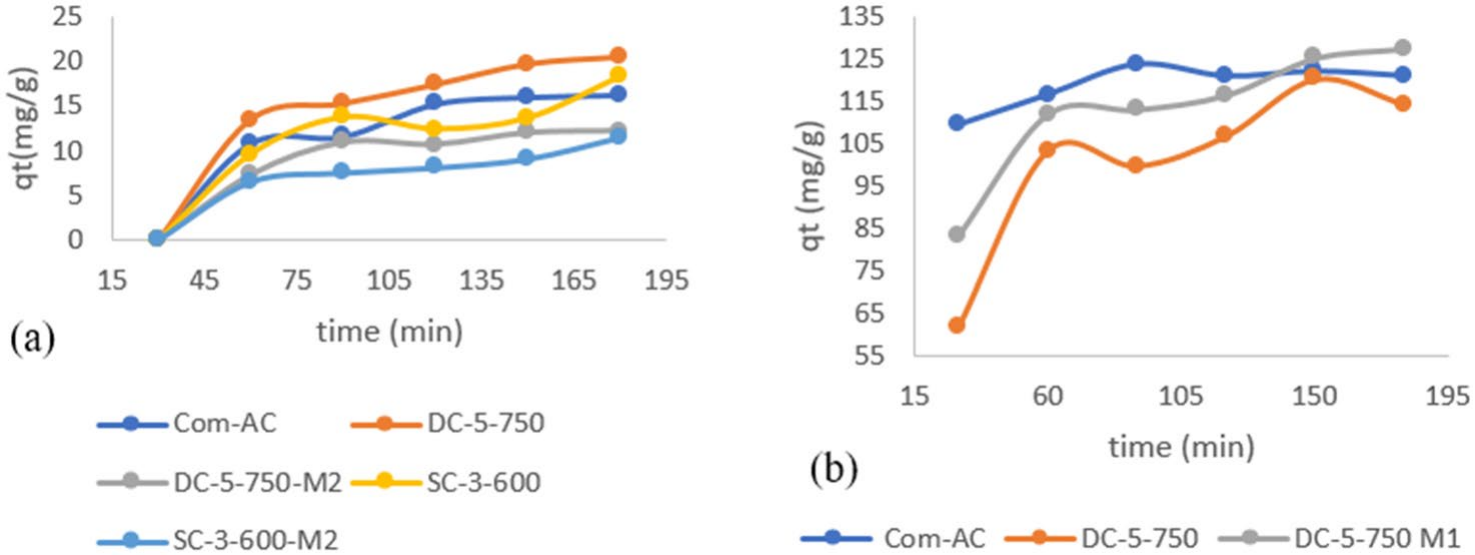
effect of time on nitrate (**a**) and MR (b) adsorption capacity.

Figure 16a depicts the nitrate removal trend as a function of time. Initially, all adsorbent adsorption capacities were negligible prior to 30 min of contact time possibly due to film diffusion resistance (external diffusion), and thereafter rose significantly for Com-AC (15.1 mg/g), DC-5-750 (17.5 mg/g), and DC-5-750M1 (10.72 mg/g).

Figure 16.b depicts the changes in dye adsorption capacity with contact time. Com-Ac adsorption capacities increased slightly from 109.63 mg/g at 30 min to 123.868 mg/g at 90 min and 121.107 mg/g at 180 min, while DC-5-750 and DC-5-750M1 adsorption capacities increased from 83.08 mg/g and 113 mg/g at 30 min to 113.126 mg/g and 123.8 mg/g at 90 min and 114.36 mg/g and 127mg/g at 180 min.

The rapid increase in adsorption capability at the start of the process could be attributed to the abundance of adsorption sites^26,49^.

**Figure 17:**
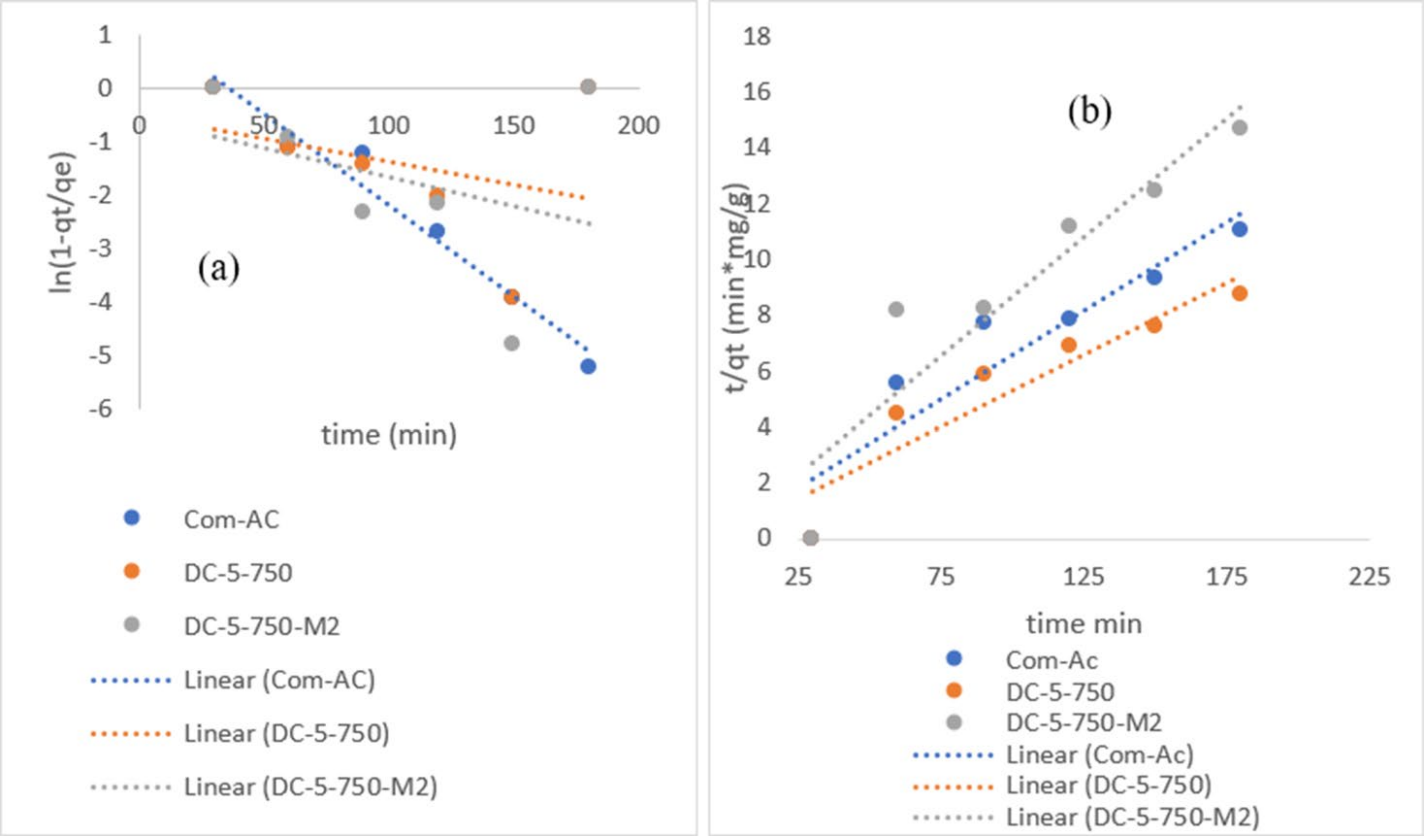
nitrate PSO (**a**) and PFO (**b**) kinetic models plots.

Nitrate pseudo first order (PFO) and Pseudo second order (PSO) plots are recorded in Fig. 17a, b and the corresponding parameters are recorded in Table 6. From the results in Fig. 17a, b Com-AC fitted better the PFO (R^2 ^= 0.9693) than PSO (R^2 ^= 0.8569) kinetic model, while other sorbents fitted better PSO kinetic model albeit lower coefficient correlation (R^2^) 0.7859 and 0.793 for SC-3-600 and SC-3-600 M1 respectively.

**Figure 18:**
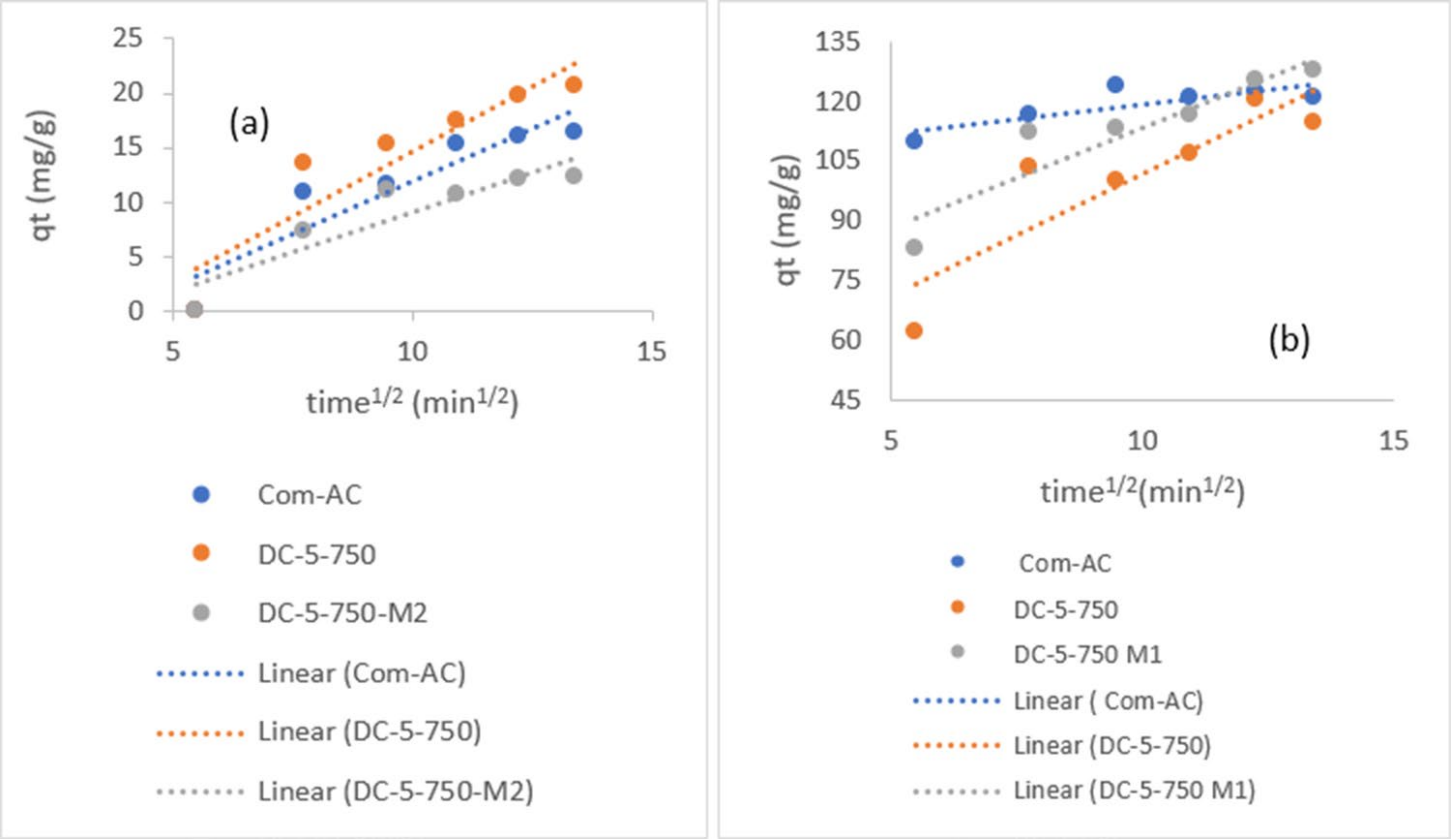
intraparticle model diffusion of nitrate (**a**) and MR (**b**) adsorption.

Figure 18 depicts intraparticle diffusion model line plots for nitrate adsorption that did not pass through the axis origin, indicating that the nitrate removal mechanism was not solely controlled by the intraparticle diffusion model. Observations of similar nitrate removal were recorded by others^25,50^.

**Figure 19:**
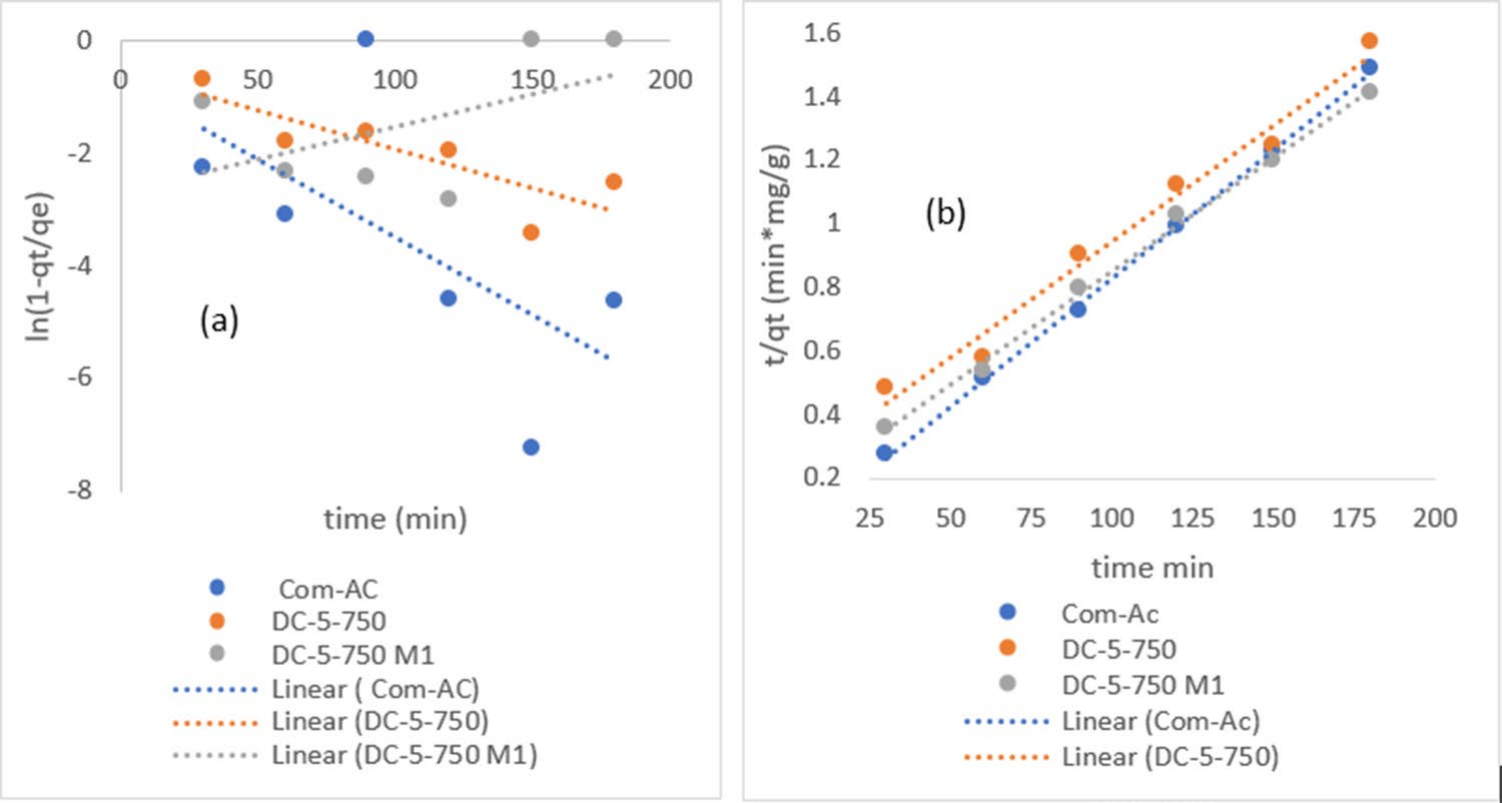
MR PFO (**a**) and PSO (**b**) kinetic model.

Nitrate PFO and PSO parameters are shown in Table 7. It was noticed that Com-AC fitted better PFO (R^2 ^= 0.9693) than PSO (R^2 ^= 0.8569) kinetic model, whereas other sorbents fit better PSO kinetic model, albeit with lower coefficient correlation (R^2^) 0.7859 and 0.793 for SC-3-600 and SC-3-600 M1, respectively. The plot of MR PFO and PSO kinetic models are depicted in Fig. 19a, b. .The MR adsorption process could not be depicted using the PFO kinetics model due to insufficient linearity fitting induced by the inherent equation formula where q_e_ (adsorption capacity) is concomitantly fitting data and determining value. The parameters for parameters value of MR PFO and PSO kinetic and intraparticle model are presented in Table 8.

**Table 7:** Parameters of PFO, PSO kinetics and intraparticle diffusion nitrate model.

	Com-AC	DC-5-750	DC-5-750M1	SC-3-600	SC-3-600M1
**Pseudo first-order**					
q_e_ (mg/g)	3.49	N/A	N/A	N/A	N/A
K_1_ (min^−1^)	0.0344	N/A	N/A	N/A	N/A
R^2^	0.9683	0.108	0.1128	0.1543	0.1186
**Pseudo second order**					
q_e_ (mg/g)	15.748	19.342	11.764	15.797	10.277
K_2_ (g mg^−1^ min^−1^)	0.016	0.016	0.037	0.0072	0.0091
R^2^	0.8569	0.8698	0.8663	0.7859	0.793
**Interparticle diffusion**					
K_id_ (g mg^−1^. min^−0.5^)	2.327	2.367	1.451	1.937	1.2479
C	− 14.032	− 8.966	− 5.475	− 7.8526	− 5.159
R^2^	0.8953	0.8525	0.8287	0.8446	0.8827

**Table 8:** Parameters value of MR PFO and PSO kinetic and intraparticle model.

	Com-AC	DC-5-750	DC-5-750M1	DC-7-900	DC-7-900M1
**Pseudo first-order**					
q_e_ (mg/g)	NA	NA	NA	NA	NA
K_1_(min^−1^)	NA	NA	NA	NA	NA
R^2^	0.3935	0.7036	0.2932	0.619	0.5439
**Pseudo second order**					
q_e_ (mg/g)	123.456	136.986	140.84	131.578	136.98
K_2_ (g*mg^−1^*min^−1^)	2.8 × 10^−3^	2.4 × 10^−4^	3.5 × 10^−4^	1.8 × 10^−4^	2.1 × 10^−4^
R^2^	0.9815	0.9815	0.9966	0.9831	0.9779
**Interparticle diffusion**					
K_id_	1.4188	6.1545	5.045	6.165	5.802
C	105.07	40.275	63.086	27.736	40.181
R^2^	0.643	0.7726	0.8644	0.978	0.9509

### pH dependence

**Figure 20:**
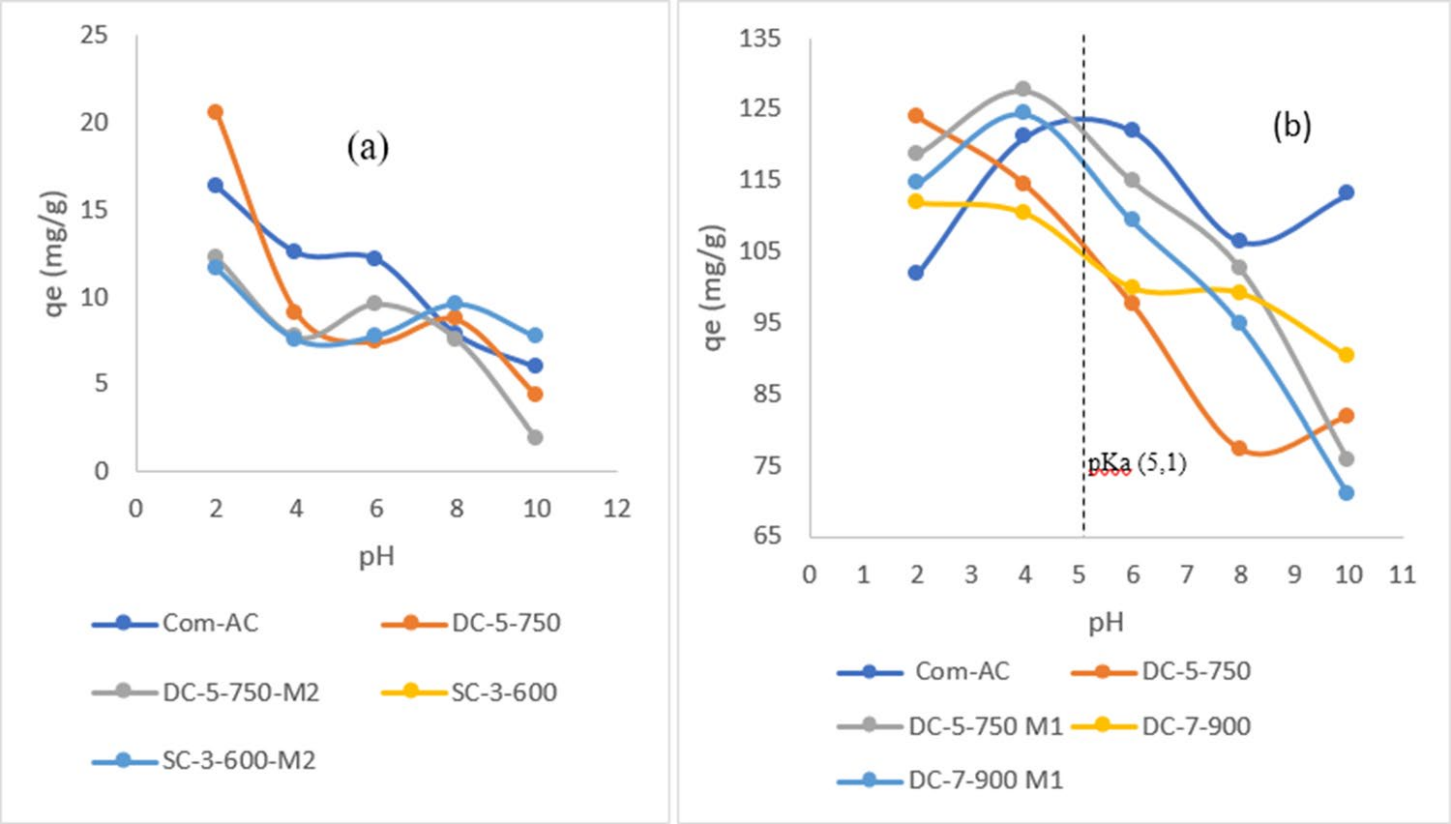
Effect of pH solution on nitrate (**a**) and MR (**b**) adsorption.

The effect of pH was measured by varying the pH solution from 2 to 10, as shown in Fig. 20. The process was pH dependable, as evidenced by adsorption decrease with pH increasing, in the case of DC-5-750 (pH_pzc _= 6.6) from 20.56 mg/g at pH 2, to 6.24 mg/g at pH 6 and 4.2 mg/g at pH 10, Com-AC (pH_pzc _= 10.3) and DC-5-750M2 (pH_pzc _= 3.1). At pH 2, adsorption potential was 16.32 mg/g and 12.24 mg/g, respectively, at pH 6, 11.12 mg/g and 9.6 mg/g, respectively, and at pH 10.6 mg/g and 1.8 mg/g. This pattern was more likely caused by: (1) favorable conditions of nitrate removal accentuated by electrostatic attraction as adsorbent surface bears positive charge at lower pH. (2) The presence of competition between nitrate ions and hydroxyl ions in basic solution, as also stated in other works using carbon-based activated carbon content^47,48,51^. In addition to the above-mentioned justifications, it may be further hypothesized that the underperformance of oxidized SBAC is due to the introduction of an acidic surface functional group; in the case of deprotonation, if pH > pHpzc, more binding sites for cationic sorbate are created on the surface than if it was an unoxidized adsorbent^44,52^.

Taking into account that MR is negative if pH>pKa and positive^26^, the introduction of acidic functional groups caused a shift in pH_pzc_, from neutral 6.6 (DC-5-750) to acidic after oxidation 3.1 (DC-5-750-M1) and Com-AC was basic 10.2, surface functional group deprotonated when pH_pzc_ < pH and adsorbents surface becomes negatively charged^53^. In contrast to SBAC, Com-AC (pH_pzc _= 10.2) has a wider range where the surface's net charge is positive. Adsorption of MR with changed SBAC, which had a lower pH_pzc_ than unoxidized, was more pH dependable due to electrostatic attraction between the adsorbent negatively charge and positive MR below pH4^26^; at pH4, the adsorption potential of DC5-750M1 and DC-7-900M1 was 127.634mg/g and 117.176 mg/g, respectively, from pH 6, pH 8, and pH 10. Adsorption capacity decreased at pH6 from 97.488mg/g for DC-5-750M1 and 109.278 for DC-7-900M1 to 81.854 mg/g for DC-5-750M1 and 71.028 mg/g for DC-7-900M1. The results show that for the oxidized adsorbent at pH4 the adsorption mechanism was related to electrostatic attraction and hydrophobic associated to/or hydrogen bond^54^. Similarly, the adsorption potential of unoxidized SBAC (DC-5-750 and DC-7-900) decreased from 123.868 mg/g and 111.76 mg/g at pH 2 to 81.854 mg/g and 90.32 mg/g at pH 10.

The outperformance in basic solution could be attributed to adsorption site rivalry between hydroxyl ion and MR negatively charged ions^49^. Similarly, in an acidic solution with a pH of 2, the rivalry may have been between H^+^ and positive MR^55^ or/and repulsion force between protonated adsorbent surface and MR^26^.

However, pH solution variation affected slightly the Com-AC adsorption capacity of MR, from 101.894 mg/g at pH 2 to 113.04 mg/g at pH 10, possibly because the electrostatic attraction mechanism was not very pronounced in the process because below pH 5.1 dye was charged positively and the protonated adsorbent had positive net charge, similar results were recorded on adsorption of cationic dye (Methyl blue)^56^.

The adsorption capacity increased as initial concentration increased because driving force of concentration gradient prevailed and had propensity to subjugate mass transfer resistance barrier between solid and liquid interface. Conversely, the proportion of nitrate extracted decreased due to adsorbent site saturation, so a fraction of sorbate remained in solution^14^. As the initial pollutant concentration increased, the adsorbent's dye adsorption capacity increased, but the proportion of dye removed decreased due to a stronger driving force to overcome mass transfer resistance in terms of adsorption capacity and less available adsorption^55,57^ For example, the adsorption capacity of Com-AC, DC-5-750, DC-5-750M2, and SC-3-600 at the lowest initial concentration (10 mg/l) was 5.78 mg/g, 8.50 mg/g, 6.28 mg/g, and 8.51 mg/g, respectively; at 50 mg/l it was 16.32 mg/g, 20.56 mg/g, 12.24 mg/g, and 18.32 mg/g, respectively; and at the highest initial concentration (90 mg/l 13 mg/g, 23.98 mg/g, 14.65 mg/g, and 18.778 mg/g, while the proportion of nitrate extracted displayed the opposite pattern. At the lowest initial concentration (10 mg/l), it was 27.78%, 40.87%, 30.19%, and 40.91%, and then decreased at the initial concentration of 50 mg/l to 16.32%, 20.56%, 12.4%, and 18.32%.

### Isotherms

**Figure 21:**
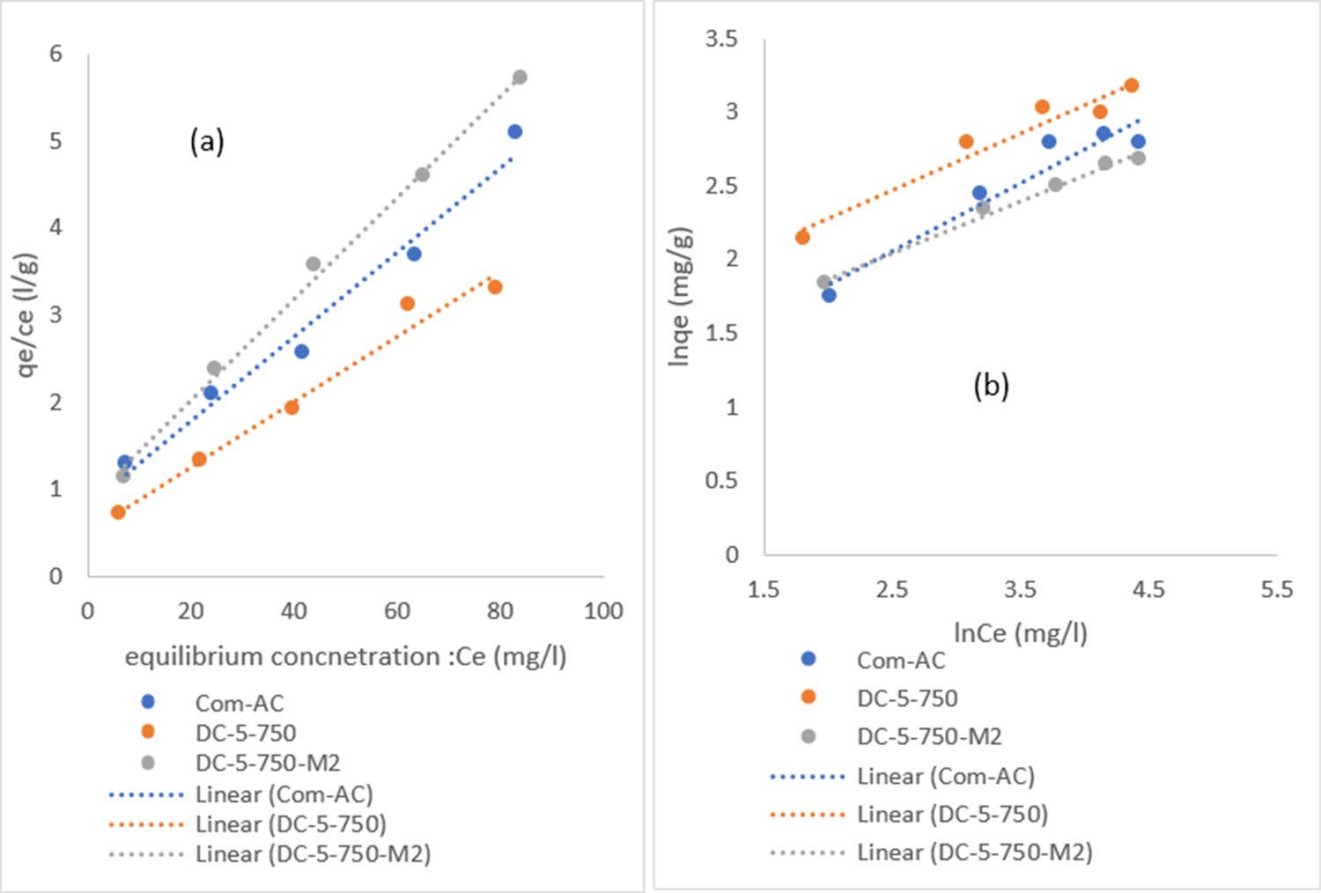
Langmuir (**a**) and Freundlich (**b**) isotherm plot of MR adsorption.

**Figure 22:**
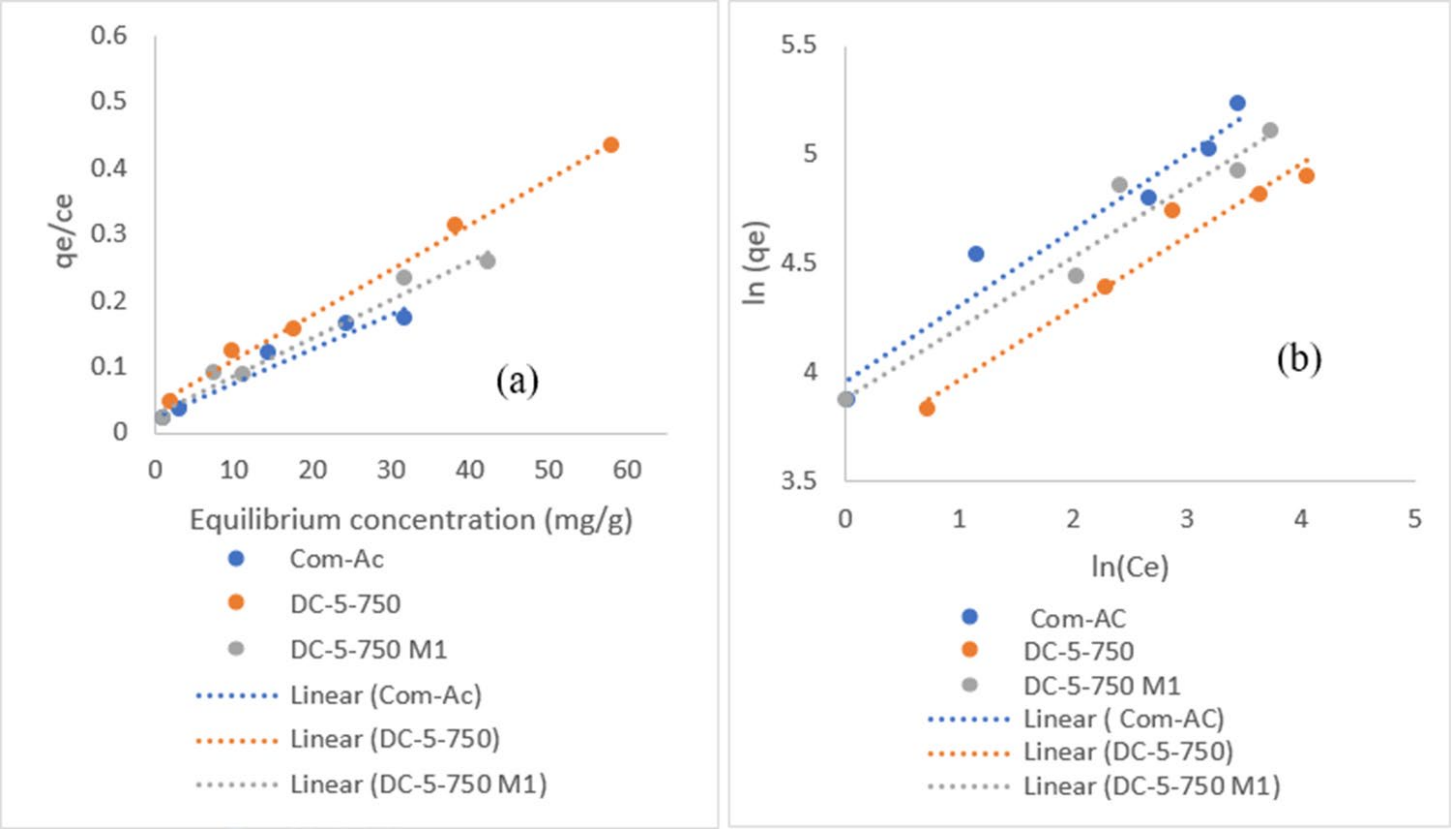
Langmuir (**a**) and Freundlich (**b**) isotherm plot of nitrate adsorption.

Figures 21a, b and 22 a, b display Langmuir and Freundlich isotherms fitted to elucidate the nitrate and MR removal processes, respectively. Tables 9 and 10 provide data on the fitting parameters. For all adsorbents, the Langmuir isotherm suit the process better with a higher R^2^ than the Freundlich model. The presence of R_L_ values between 0 and 1 suggested that nitrate and MR adsorption were favorable on all sorbent surfaces. It is worth noting, however, that as the R_L_ value reached zero, MR adsorption became more irreversible with increasing concentration^12,35^.

**Table 9:** Parameters value of Langmuir and Freundlich isotherm of MR removal.

	Com-AC	DC-5-750	DC-5-750M1	DC-7-900	DC-7-900M1
**Langmuir**					
Qm (mg/g)	196.07	147.058	175.438	136.986	156.25
K_L_ (L/mg)	0.2082	0.1645	0.1919	0.1648	0.2229
R_L_	0.1617–0.037	0.194–0.046	0.1724–0.04	0.1953–0.0463	0.1521–0.0346
R^2^	0.9515	0.9955	0.9707	0.9992	0.9967
**Freundlich**					
K_F_ (mg/g) (l/mg)^1/n^	51.997	37.765	48.443	33.794	45.141
1/n (l/mg)	0.035	0.3306	0.324	0.345	0.3199
R^2^	0.9492	0.957	0.9463	0.9242	0.9578

**Table 10:** Comparison of MR adsorption capacity with literature values.

Adsorbent type	Surface area (m^2^/g)	Solution pH	Concentration range	q_m__(mg/g)_	References
NaOH lemongrass leaves AC*	834.04	2	25–500	76.923	^6^
KOH durian seed AC	980.62	–	25–500	384.62	^49^
Iron oxidized AC	153.32	–	–	526	^26,58^
K_2_CO_3_ custard apple AC	431.05	5	80–120	171.23	
H_3_PO_4_ custard apple AC	1065	4	80–120	435.25	
Woody biochar	4.96	–	–	156.25	^30^
COM-AC	630.209	4	25–125	196.07	This work
DC-5-750	312.79	4	25–125	147.058	
DC-5-750M1	305.51	4	25–125	175.438	

*AC* Activated carbon.

Nitrate Langmuir and Freundlich isotherms were used to investigate the nitrate removal process. The findings are presented in Table 11.

**Table 11:** Value of isotherms parameters of nitrate removal.

	Com-AC	DC-5-750	DC-5-750M1	DC-7-900	DC-7-900M1
**Langmuir**					
Qm (mg/g)	20.618	26.737	17.064	21.276	17.513
K_L_ (L/mg)	0.8165	0.5165	0.8561	0.5437	1.2918
R_L_	0.109–0.0134	0.1622–0.021	0.1045–0.0128	0.1553–0.02	0.0718–0.0085
R^2^	0.9759	0.9772	0.9959	0.9813	0.9967
**Freundlich**					
K_F_ (mg/g) (l/mg)^1/n^	2.454	4.4942	3.2072	4.744	1.8323
1/n (l/mg)	0.4648	0.3873	0.3552	0.322	0.4724
R^2^	0.9264	0.9455	0.9901	0.9317	0.9583

As predicted, the Langmuir isotherm described the process better than the Freundlich model, with a greater R^2^ for all adsorbents involved on a monolayer surface. The value of R_L_ between 0 and 1 indicated that both sorbate adsorption was favourable on all sorbent surfaces of the Langmuir isotherm model, it is regarded as unfavourable if R_L _> 1^36^, for Freundlich model adsorption intensity (1/n) value was less than 0.5 in all cases, indicating that sorbate was easily adsorbed, it is hardly adsorbed if 1/n > 2^42^.

It is worth noting that MR adsorption became more irreversible with increasing concentration since the RL value approached zero at higher concentration^25,26^. The published data are also compared to the present study's nitrate adsorption capacities in Table 12.

**Table 12:** Comparison of nitrate Langmuir maximum capacity with literature.

Adsorbent type	Surface area (m^2^/g)	Solution pH	Concentration range (mg/l)	q_m__(mg/g)_	References
Sawdust AC* with KOH	768	2	8.5–510	25.499	^51^
Commercial AC	1424	2	8.5–510	19.549	
Urea treated AC	192	2	8.5–680	38.824	^8^
Thermally post- treat AC	772	2	8.5–680	25.499	
AC oxidized with CETAB*	901	7	40–200	21,51	^25^
Metal oxidized biochar	155.08	–	50–1500	32.23	^59^
ZnCl_2_ olive stone AC	1480	4	100–300	5.525	^5^
Municipal sewage sludge biochar	2.82	2	20–100	2.1127	^60^
Commercial AC	603.209	2	10–90	20.618	This work
DC-5-750	312.79	2	10–90	26.737	
DC-5-750M2	282.22	2	10–90	17.064	

AC* Activated carbon, *CETAB* cetyl trimethyl ammonium bromide.

### Ionic strength

**Figure 23:**
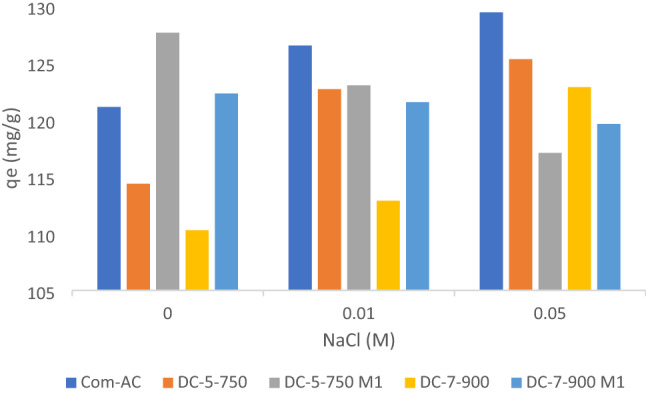
effect of ionic strength on MR uptake.

To assess the effect of ionic strength, MR was diluted in NaCl solution with a concentration of 0.01 M and 0,05M and the pH was adjusted at 4. As shown in Fig. 23, presumably Na^+^ cations competition and screening effect of Cl^-^ anions at the external surface of adsorbent^54^ caused adsorption capacity of oxidized SABC to dwindle narrowly with NaCl concentration change from 127.63 mg/g (without NaCl) to 117.08 mg/g (0.05M NaCl) for DC-5-750M1 and 122.29 mg/g (without NaCl) to 119.61 mg/g(0,05M NaCl) for DC-7-900M1, contrariwise Com-AC and unoxidized adsorbents (DC-5-750 and DC-7-900) increased with NaCl concentration augmentation from 121.08 mg/g to 129.42 mg/g, 114.43 mg/g without NaCl to 125.31 mg/g and 110.29 mg/g to 122.85 mg/g in presence of 0.05M NaCl respectively, adsorption capacity upward trend could have been imputed arguably to dye aggregation drove by salt ions force^61^.

### Environmental consideration: toxicity characteristic leaching procedure

The toxicity contaminant leaching procedure was carried out as described elsewhere^39,62^, and the element concentrations were determined using a Perkin-Elmer AA spectrometer. Table 13 shows the results of the TCLP test; in general, the concentration of leachable heavy metal in the pyrolyzed adsorbent was lower than the precursor due to the higher thermal stability of heavy metal acquired through pyrolysis^62^.

**Table 13:** Heavy metal concentration leached from precursors and sorbents.

	Elements (mg/l)							
	Cu	Zn	Pb	Ni	Co	Cr	Fe	Mn
D	3.534	0.0498	18.99	0.329	0.0061	0.000	0.670	0.000
S	3.228	0.0970	25.11	0.000	0.332	0.000	0.145	0.006
Discard Coal	3.520	0.2773	27.46	0.000	0.336	0.000	0.000	0.005
Com-AC	0.897	0.1238	0.58	0.073	0.713	0.575	0.000	0.068
SC-3-600	3.154	0.1247	24.93	4.064	0.326	0.000	0.000	0.007
SC-5-900	1.922	0.2474	20.09	4.236	0.752	1.555	17.092	2.798
DC-5-750	3.191	0.0379	24.99	0.000	0.279	0.000	0.000	0.003
DC-7-900	3.306	0.0294	24.81	0.000	0.489	0.000	0.000	0.000

However, after pyrolysis, SC-5-900 released more heavy metals (Fe, Cr, Co, and Ni) than its precursors. This may be due to the disintegration of some stable inorganic minerals (primarily silicate and carbonate) from sludge during pyrolysis with temperature augmentation, which caused the liberation of the fixed metals from the lattice^17^.

## Conclusion

Two kinds of sewage sludge were used in this paper to create low-cost adsorbents: D, which was collected during the dissolved air flotation stage, and S, a combination of primary and secondary sludge from the digestion and dewatering phases. After mixing the sewage sludge with waste coal, it was activated with KOH and oxidized with APS. We evaluated and compared the capacity of the synthesized adsorbents to remove nitrate and MR to that of commercial activated carbon. The oxidation with APS (1) had a greater negative effect on the textural properties of adsorbent derived from sludge S than on those derived from sludge D, (2) had a negligible effect on the organic composition as revealed by ultimate analysis, and (3) induced the introduction of acidic functional groups as revealed by FT-IR and Raman spectroscopy, respectively. The adsorption capacity of adsorbents increased with time and initial contamination concentrations. The nitrate and MR removal processes followed the pseudo-second order kinetic model and the Langmuir isotherm rather well. Nitrate and MR adsorption capabilities were greater at pH = 2 and pH = 4, respectively.

## Data Availability

Some of the data will be made available upon the journal acceptance.
